# Genomic and cytogenetic analyses reveal satellite repeat signature in allotetraploid okra (*Abelmoschus esculentus*)

**DOI:** 10.1186/s12870-024-04739-9

**Published:** 2024-01-25

**Authors:** Jiarui Liu, Xinyi Lin, Xiaojie Wang, Liqing Feng, Shixin Zhu, Runmeng Tian, Jingping Fang, Aifen Tao, Pingping Fang, Jianmin Qi, Liwu Zhang, Yongji Huang, Jiantang Xu

**Affiliations:** 1 Scientific Observing and Experimental Station of Southeastern kenaf & jute, Ministry of Agriculture and Rural Affairs of the People’s Republic of China, Key Laboratory of Ministry of Education for Genetics, Breeding and Multiple Utilization of Crops, Fujian Provincial Key Laboratory of Crop Breeding by Design, National Engineering Research Center for Sugarcane, College of Agriculture, Fujian Agriculture and Forestry University, Fuzhou, 350002 China; 2 College of Life Science, Fujian Agriculture and Forestry University, Fuzhou, 350002 China; 3 College of Life Science, Fujian Normal University, Fuzhou, 350117 China; 4 Ministerial and Provincial Joint Innovation Centre for Safety Production of Cross-Strait Crops, College of Geography and Oceanography, Minjiang University, Fuzhou, 350108 China

**Keywords:** Satellite repeat, rDNA, Intergenic spacer region, Allotetraploid, *Abelmoschus esculentus*, Fluorescence in situ hybridization

## Abstract

**Background:**

Satellite repeats are one of the most rapidly evolving components in eukaryotic genomes and play vital roles in genome regulation, genome evolution, and speciation. As a consequence, the composition, abundance and chromosome distribution of satellite repeats often exhibit variability across various species, genome, and even individual chromosomes. However, we know little about the satellite repeat evolution in allopolyploid genomes.

**Results:**

In this study, we investigated the satellite repeat signature in five okra (*Abelmoschus esculentus*) accessions using genomic and cytogenetic methods. In each of the five accessions, we identified eight satellite repeats, which exhibited a significant level of intraspecific conservation. Through fluorescence in situ hybridization (FISH) experiments, we observed that the satellite repeats generated multiple signals and exhibited variations in copy number across chromosomes. Intriguingly, we found that five satellite repeats were interspersed with centromeric retrotransposons, signifying their involvement in centromeric satellite repeat identity. We confirmed subgenome-biased amplification patterns of these satellite repeats through existing genome assemblies or dual-color FISH, indicating their distinct dynamic evolution in the allotetraploid okra subgenome. Moreover, we observed the presence of multiple chromosomes harboring the 35 S rDNA loci, alongside another chromosomal pair carrying the 5 S rDNA loci in okra using FISH assay. Remarkably, the intensity of 35 S rDNA hybridization signals varied among chromosomes, with the signals predominantly localized within regions of relatively weak DAPI staining, associated with GC-rich heterochromatin regions. Finally, we observed a similar localization pattern between 35 S rDNA and three satellite repeats with high GC content and confirmed their origin in the intergenic spacer region of the 35 S rDNA.

**Conclusions:**

Our findings uncover a unique satellite repeat signature in the allotetraploid okra, contributing to our understanding of the composition, abundance, and chromosomal distribution of satellite repeats in allopolyploid genomes, further enriching our understanding of their evolutionary dynamics in complex allopolyploid genomes.

**Supplementary Information:**

The online version contains supplementary material available at 10.1186/s12870-024-04739-9.

## Background

Okra (*Abelmoschus esculentus*), a member of the family *Malvaceae*, is widely cultivated in tropical and subtropical regions worldwide [1]. Due to its rich nutritional elements and significant dietary fiber content, okra has been highly favored as a horticultural crop [2, 3]. Additionally, it is rich in magnesium, iron, polyphenols, and folate, which contribute to its medicinal properties, including antioxidant, anticancer and antidiabetic activities [4, 5]. The formation of *A. esculentus* might have occurred through hybridization between *A. tuberculatus*, an indigenous species from Northern India, and *A. ficulneus*, a species from East Africa. This hybridization event, followed by chromosome doubling, led to the formation of an allopolyploid *Abelmoschus* hybrid [6]. Recent finding from genome assembly of an okra variety ‘Wufu’ has provided evidence supporting its identity as an allotetraploid [7].

Satellite repeats, initially regarded as junk DNA due to their non-coding nature, have increasingly been found to have functional significance [8, 9]. Accumulating evidences suggest that they play crucial roles in heterochromatin domain formation, accurate chromosome segregation, and chromatin-mediated regulation of gene expression [10–12]. Satellite repeats are predominantly abundant in heterochromatin regions of eukaryotic chromosomes, including centromeres, pericentromeres and subtelomeres [11]. They are arranged in long arrays of highly similar head-to-tail tandemly repeated units (monomers). Intriguingly, satellite repeats are one of the most rapidly evolving parts of the genome, resulting in extreme diversity in monomer size, genomic abundance, nucleotide sequences, and chromosomal distribution, even among closely related species [8]. Another significant region of tandem repeat sequences within nuclear DNA is the ribosomal DNA (rDNA). It comprises two distinct gene classes, the major cluster 35 S rDNA and the minor cluster 5 S rDNA. The 35 S rDNA includes genes for 18 S, 5.8 S, and 28 S rRNA, along with two internal transcribed spacers (ITS1 and ITS2) and intergenic spacers (IGS). On the other hand, the 5 S rDNA gene consists of a gene transcription region and a non-transcribed spacer (NTS) [13]. Although rDNA is among the most evolutionarily conserved DNA sequences, the intergenic spacer sequences are less subject to functional constraints and evolve at higher rates [14]. Consequently, these intergenic spacer sequences are commonly employed for phylogenetic inference at low taxonomic levels due to their high substitution rates and low intragenomic sequence heterogeneity.

Allopolyploid species serve as excellent models for investigating the evolutionary trajectory of satellite repeats [15, 16]. Diverse satellite repeat variants may arise from a common ancestral sequence and undergo rapid and dynamic evolution, including the nucleotide sequence variation and genomic abundance change, particularly allopolyploidization, which combines different genomes of distinct progenitor species [17]. For instance, within allotetraploid *Chenopodium quinoa*, several satellite repeat subfamilies have swiftly adapted to become subgenome-specific and have displayed varying genomic abundance patterns [15]. Although the current okra reference genome was released in 2023, satellite repeats have yet to be conclusively identified [7]. Given the existing knowledge gap regarding satellite repeat evolution in the okra genome, the allotetraploid okra presents a potentially attractive for gaining insights into the impact of allotetraploidization on the evolution of satellite repeats in the okra genome.

Over a decade ago, satellite repeats were frequently underrepresented or even absent in genome assemblies due to their highly repetitive nature and identical or nearly identical sequences [18]. However, recent advancements in sequencing technologies, such as PacBio and Nanopore, have enabled the generation of longer reads capable of assembling large satellite arrays, ultimately allowing for the completion of telomere-to-telomere (T2T) genome assemblies [19, 20]. Despite these breakthroughs, T2T genome assembly continues to pose challenges for many species, particularly those with complex genomes. Consequently, to investigate such elusive genomic regions in the absence of T2T genome assemblies, it is crucial to employ appropriate approaches to shed light on the “dark matter” of the genome. A combination of high-throughput next-generation sequencing (NGS) and fluorescence in situ hybridization (FISH) techniques has emerged as a promising method for in-depth analysis of all components of a genome, including the chromosomal locations of satellite repeat families of interest [21]. These techniques have already proven successful in various plant species, such as *Arabidopsis thaliana*, *Brachypodium distachyon*, sugarcane, rice, potato and switchgrass [10, 22–25].

In this study, we conducted a comprehensive analysis to identify and characterize satellite repeats in five okra accessions. Eight satellite repeats were identified in each accession and FISH analysis revealed that these sequences exhibited multiple signals and variations in copy numbers across chromosomes. Interestingly, we discovered that five satellite repeat sequences were interspersed with centromeric retrotransposons. Additionally, we confirmed the biased amplification pattern within the subgenome and the chromosomal location pattern of both 35 S rDNA and 5 S rDNA. Finally, we confirmed the origin of three satellite repeat sequences with high GC content in the intergenic spacer regions of rDNA. These findings contribute to our understanding of satellite repeats and their evolutionary dynamics in the allopolyploid genome.

## Results

### Genome-wide identification revealed eight satellite repeats in five okra accessions

To identify satellite repeats in five okra accessions, we firstly sequenced their genomes using the Illumina HiSeq X platform, generating paired-end sequencing data ranging from 3.12 to 3.38 Gb in size. Subsequently, we conducted a graph-based sequence similarity clustering analysis using RepeatExplorer 2 on randomly selected 2 M pair-end reads for each accession. Based on the star or ring cluster shape and tandem arrangement, we selected a total of 40 potential satellite repeat clusters in the studies okra accessions for further analysis (Table 1).

**Table 1:** Satellite repeat characteristics in five okra accessions

Group	Satellite repeat	Monomer length (bp)	GC content (%)	Genome proportion (%)
A	AeSat-A-FO	169	31	1.20
	AeSat-A-T3	169	31	1.20
	AeSat-A-ROJ	169	31	1.10
	AeSat-A-RO1	169	31	1.20
	AeSat-A-QR	169	31	1.20
B	AeSat-B-FO	170	28	1.50
	AeSat-B-T3	170	28	1.40
	AeSat-B-ROJ	170	29	1.30
	AeSat-B-RO1	170	28	1.40
	AeSat-B-QR	170	28	1.40
C	AeSat-C-FO	170	26	0.17
	AeSat-C-T3	170	28	0.14
	AeSat-C-ROJ	170	27	0.14
	AeSat-C-RO1	170	26	0.16
	AeSat-C-QR	170	27	0.17
D	AeSat-D-FO	170	29	0.26
	AeSat-D-T3	170	28	0.30
	AeSat-D-ROJ	170	28	0.27
	AeSat-D-RO1	170	28	0.29
	AeSat-D-QR	170	28	0.31
E	AeSat-E-FO	99	48	0.92
	AeSat-E-T3	99	52	0.81
	AeSat-E-ROJ	99	52	1.20
	AeSat-E-RO1	99	48	0.90
	AeSat-E-QR	99	52	0.91
F	AeSat-F-FO	202	54	0.21
	AeSat-F-T3	202	54	0.26
	AeSat-F-ROJ	202	54	0.30
	AeSat-F-RO1	202	54	0.21
	AeSat-F-QR	202	54	0.27
G	AeSat-G-FO	136	68	0.53
	AeSat-G-T3	136	68	0.56
	AeSat-G-ROJ	136	68	0.88
	AeSat-G-RO1	136	68	0.61
	AeSat-G-QR	136	68	0.69
H	AeSat-H-FO	43	67	0.53
	AeSat-H-T3	43	67	0.56
	AeSat-H-ROJ	43	67	0.88
	AeSat-H-RO1	43	67	0.61
	AeSat-H-QR	43	67	0.69

**Fig. 1:**
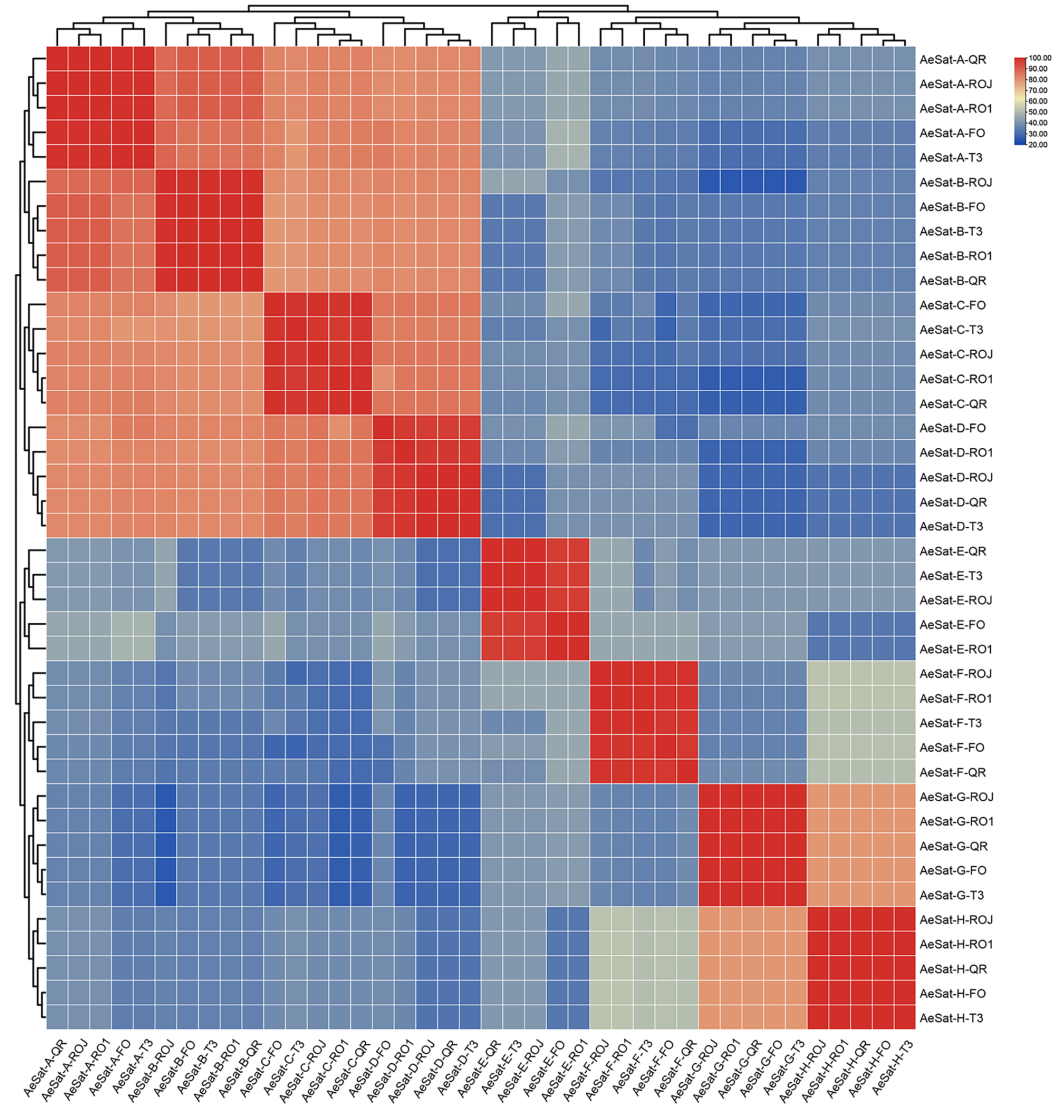
A heatmap depicting the sequence similarity of satellite repeats in five okra accessions. Satellite repeats that group together had the highest similarity indexes between them. Color scale: warmer (red) color indicated more similar, while cooler (blue) color indicated more different satellite repeats in the pairs compared

These sequences were classified into eight distinct groups based on their sequence similarities, namely AeSat-A, AeSat-B, AeSat-C, AeSat-D, AeSat-E, AeSat-F, AeSat-G and AeSat-H (Fig. 1 and S1). Within each group, we observed a high level of sequence similarity among the sequences from the five okra accessions, ranging from 97.0 to 100% (Table 1), indicating a significant level of intraspecific conservation. Interestingly, the groups AeSat-A, AeSat-B, AeSat-C and AeSat-D exhibited similar sequence characteristics (Table 1), such as higher sequence similarity (79.1–90.6%), sequence lengths approximating the size of a mononucleosome (169–170 bp), and low GC content (26–32%). These shared sequence features suggest that they have evolved from a common ancestral sequence. However, the genomic abundance of these four groups differed (Table 1). Specifically, AeSat-B had the highest genomic proportion, ranging from 1.3 to 1.5%, followed by AeSat-A (1.10–1.20%) and AeSat-D (0.27–0.31%) as the second and third most abundant, while AeSat-C had the lowest genomic abundance (0.14–0.17%). The monomer in the AeSat-E group had 99 bp in length, GC content ranging from 48 to 52%, and genomic abundance between 0.81% and 1.20% (Table 1). Additionally, the AeSat-F group had the longest monomer length (202 bp), moderate GC content (54%), and genomic abundance ranging from 0.21 to 0.30%. The AeSat-G and AeSat-H groups were GC-rich, with monomer lengths of 136 bp and 43 bp, and genomic abundance ranging from 0.53 to 0.88% for both groups (Table 1).

### Cytological assays confirmed the chromosome distribution pattern of eight satellite repeats across five accessions

**Fig. 2:**
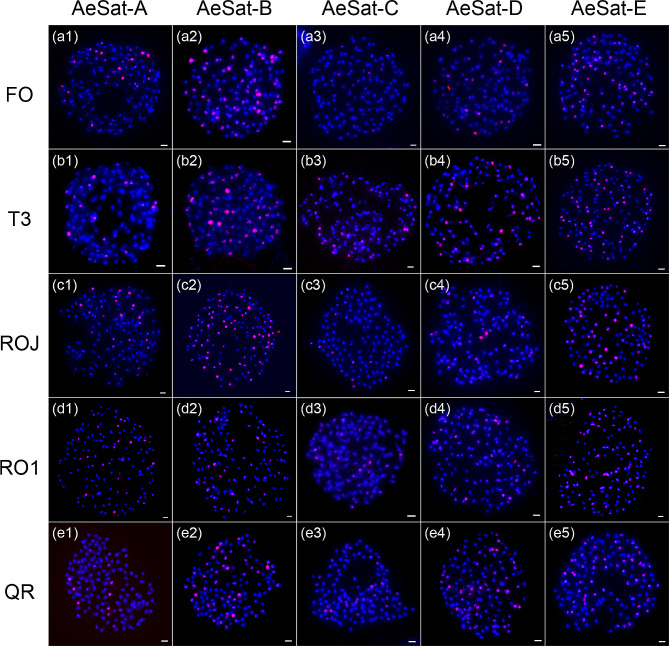
FISH mapping of satellite repeats AeSat-A, AeSat-B, AeSat-C, AeSat-D and AeSat-E in five okra accessions. DAPI-stained mitotic chromosomes are shown in blue, and the signals of five satellite repeat probes are shown in red. FISH mapping of repeats AeSat-A, AeSat-B, AeSat-C, AeSat-D and AeSat-E in FO (a1-a5),T3 (b1-b5), ROJ (c1-c5), RO1 (d1-d5) and QR (e1-e5). The signal loci of these satellite repeats exhibit variability in five okra accessions. Scale bars: 1 μm

**Fig. 3:**
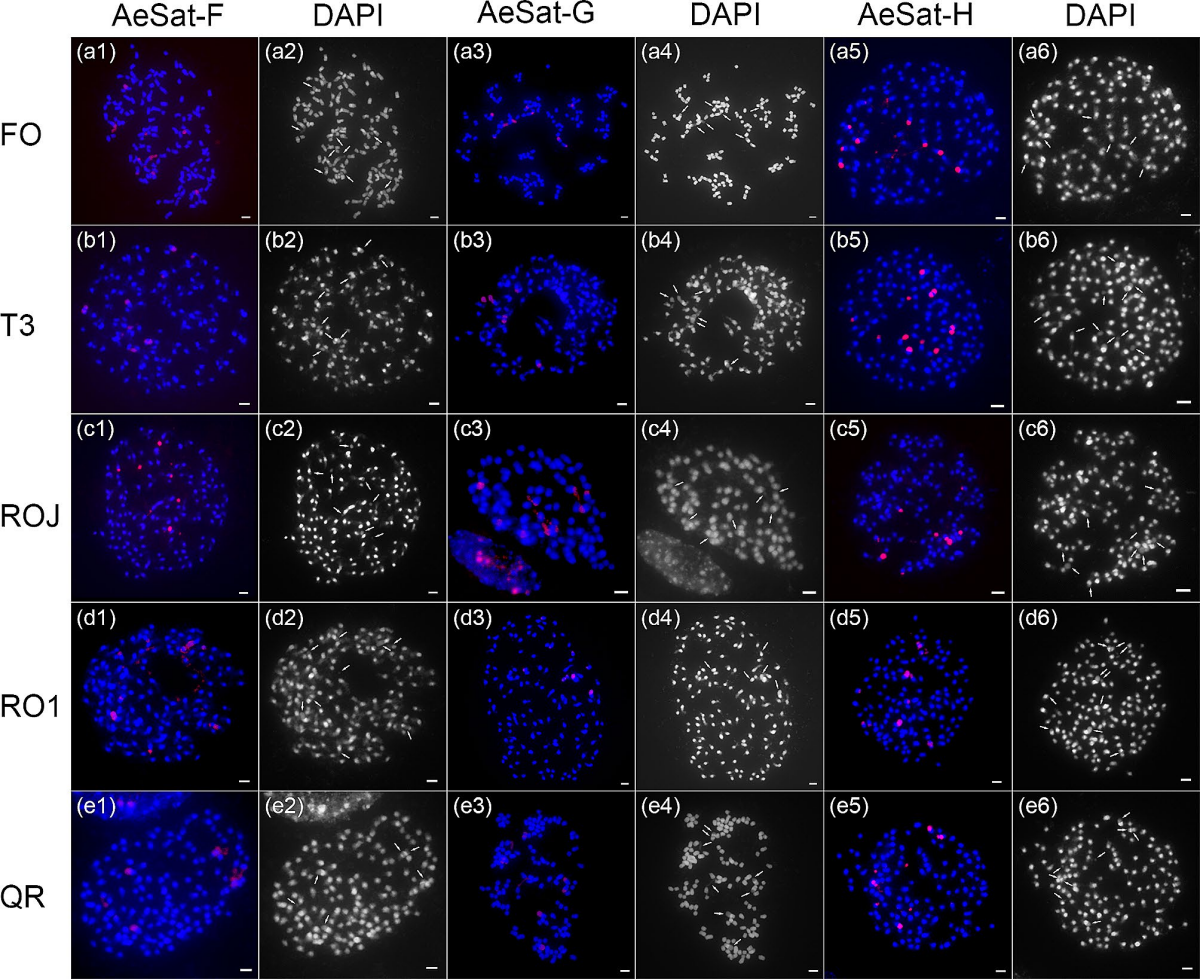
FISH mapping of satellite repeats AeSat-F, AeSat-G and AeSat-H in five okra accessions. DAPI-stained mitotic chromosomes are shown in blue and grey. The signals of three satellite repeats are shown in red. FISH mapping of repeats AeSat-F, AeSat-G and AeSat-H in FO (a1-a6), T3 (b1-b6), ROJ (c1-c6), RO1 (d1-d6) and QR (e1-e6). These signals were primarily detected in areas exhibiting low DAPI fluorescence. Scale bars: 1 μm

To ascertain the chromosome distribution of de novo identified satellite repeats, we hybridized each candidate satellite repeat probe from the eight groups to the metaphase chromosomes of the five okra accessions. FISH analyses showed that all the satellite repeat probes produced multiple signals in all of the studies accessions, confirming their identity as satellite repeats (Figs. 2 and 3). Furthermore, we consistently observed clear variations in signal intensities between the chromosomes for these probes (Figs. 2 and 3), which reflect their copy number variations across different chromosomes.

Regarding the signal number, AeSat-B and AeSat-E were detected in 64 chromosomes in all of the studied accessions. For AeSat-A, 29 signal loci were observed in the four accessions, except for the QR accession, which had 20 signal loci. Polymorphism in the number of AeSat-D signal loci was observed among these accessions, with 27 loci in FO, 53 loci in T3, 42 loci in ROJ, 60 loci in RO1, and 100 loci in QR. The number of signals in AeSat-C varied among the four okra accessions, with 27, 53, 42, and 60 signals for FO, T3, ROJ, and RO1, respectively. However, the number of signals in QR (100 loci) was more than that in the other four accessions (Fig. 2). Therefore, despite the sequence conservation observed among various accessions, the signal loci of these satellite repeats display polymorphism.

The satellite repeats, AeSat-F, AeSat-G and AeSat-H, exhibit a similar pattern of multiple signal localization (Fig. 3). Notably, these signals are predominantly observed in regions with relatively weak DAPI staining. DAPI is a fluorescent dye known for its strong affinity to AT-rich regions in DNA, while it exhibits weaker binding to GC-rich heterochromatic regions [26]. This observation is consistent with the high GC content characteristic of these three satellite repeats.

### Five centromere-associated satellite repeats exhibit distribution bias within the two subgenomes

**Fig. 4:**
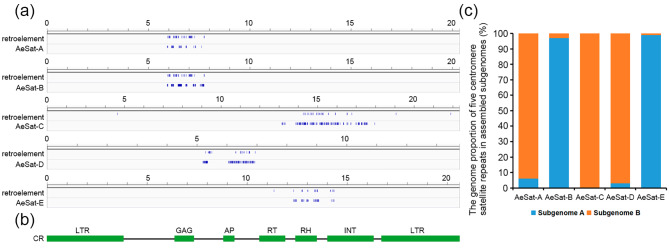
Characterization of genomic distributions of five satellite repeats in an allotetraploid okra variety ‘Wufu’. (**a**) Diagrams illustrate the distributions of a retroelement sequence and satellite repeats AeSat-A, AeSat-B, AeSat-C, AeSat-D and AeSat-E in assembled genomes. (**b**) The annotation of the retroelement protein domains in REXdb protein database. (**c**) The genome proportion of AeSat-A, AeSat-B, AeSat-C, AeSat-D and AeSat-E in assembled subgenomes

Recently, the genome assembly of an allotetraploid okra variety ‘Wufu’ has been publicly released, allowing us to analyze the precise distribution locations of these satellite repeats on chromosomes. Upon alignment with the assembled okra genome, we observed that five distinct satellite repeats (namely AeSat-A to AeSat-E) were interspersed with a retroelement sequence (Fig. 4a). To assign the retroelement to a specific retroelement subfamily, the retroelement sequence was then annotated to REXdb protein database using BLAST based on the similarity to conserved protein domains database. A query of this retroelement in the protein database disclosed that it bore all the genes required for transposition (Fig. 4b), indicating that it is an autonomous retrotransposon. We unequivocally assigned this okra retrotransposon to the CR-type chromoviruses (Fig. 4b), often marked by a preferential accumulation pattern within plant centromeres, belonging to the Ty3/gypsy chromovirus lineage [19, 20, 23–29]. Hence, this observation indicates their classification as centromeric satellite repeats. Additionally, we have also observed that AeSat-B and AeSat-E predominantly colonize the A subgenome, whereas AeSat-A, AeSat-C and AeSat-D display a distinct preference for distribution within the B subgenome (Fig. 4c).

**Fig. 5:**
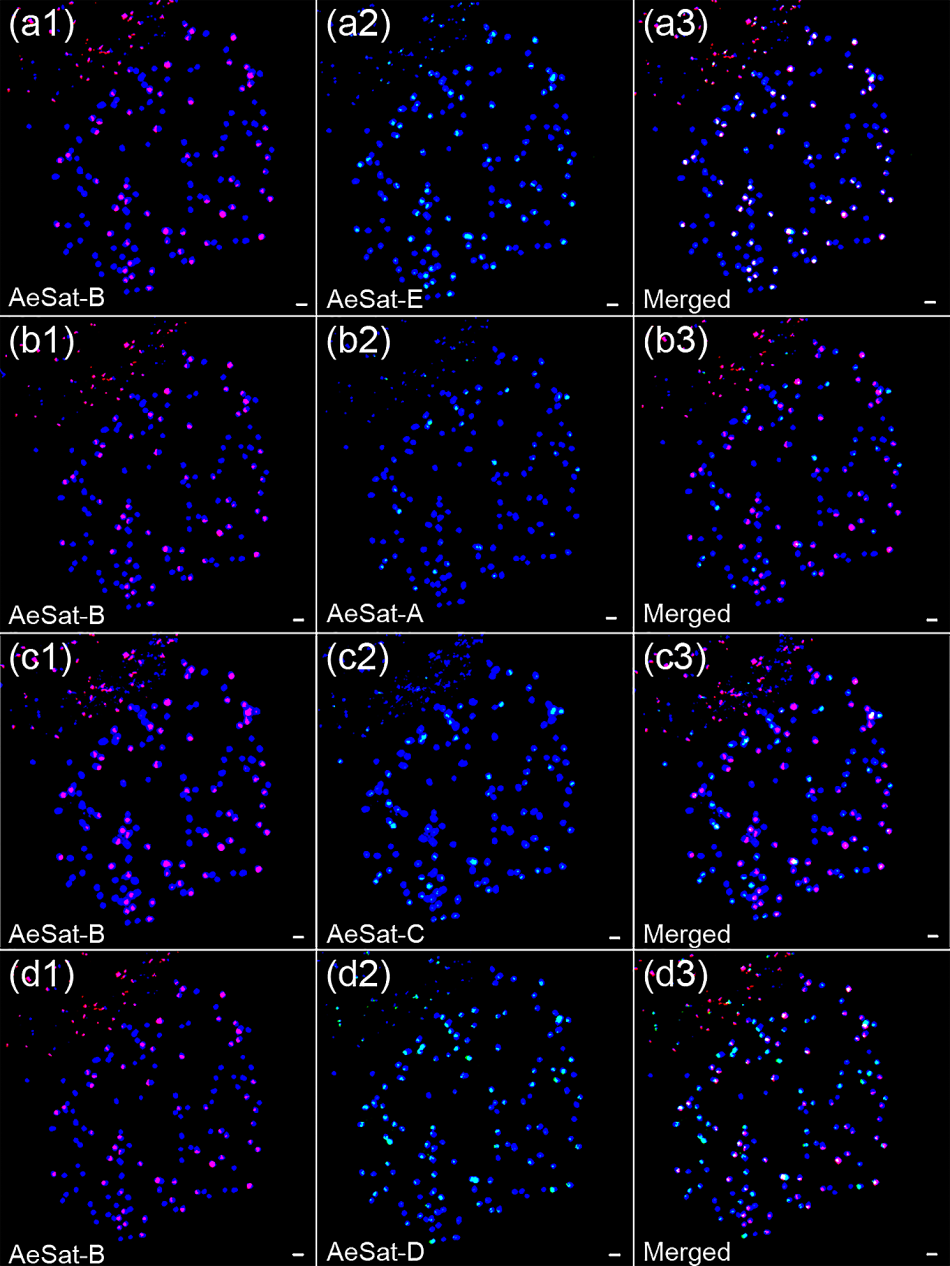
FISH localization of five centromeric satellite repeats on mitotic metaphase chromosomes of RO1 accession. (**a**) Dual-color FISH of AeSat-B (red) and AeSat-E (green), showing colocalization of these two satellite repeats on the chromosomes of RO1; (**b**) Dual-color FISH of AeSat-B (red) and AeSat-A (green), showing non-colocalization of these two satellite repeats on the chromosomes of RO1; (**c**) Dual-color FISH of AeSat-B (red) and AeSat-C (green), showing non-colocalization of these two satellite repeats except on one pair of chromosomes of RO1; (**d**) Dual-color FISH of AeSat-B (red) and AeSat-D (green), showing colocalization of these two satellite repeats on a few chromosomes of RO1. Thus, five centromeric satellite repeats exhibit distribution bias within the two subgenomes in RO1 accession. Scale bars: 1 μm

To determine the chromosomal distribution patterns of these satellite repeats in five okra accessions, we performed dual-color FISH analysis on their metaphase chromosome spreads. Dual-probe FISH using AeSat-B and AeSat-E revealed the overlapped signals from these two satellite repeats in RO1 (Fig. 5). Similar chromosomal distribution patterns were observed in the other four okra accessions (Figures S2-S5). In contrast, the signals of AeSat-B did not co-localize with those of AeSat-A and AeSat-C except on one pair of chromosomes of all five analyzed accessions (Fig. 5 and S2-S5). Furthermore, AeSat-B signals only co-localized with AeSat-D signals on a few chromosomes (Fig. 5 and S2-S5). These results are consistent with the analysis results of the okra variety ‘Wufu’ genome assembly, suggesting that these sequences are highly conserved in the chromosome distribution patterns of different okra accessions (Fig. 4b).

### 35 S rDNA mainly resides in AT-rich heterochromatin regions of both subgenomes, while 5 S rDNA predominantly localizes in subgenome A

To determine the sequence conservation of the intergenic regions of 5 S rDNA and 35 S rDNA (herein ITS1 + 5.8 S + ITS2 sequence of 35 S rDNA and the 5 S rDNA + NTS sequence) in five okra accessions, we used DNAMAN8 software to perform multiple sequence alignments on the sequences of the five okra accessions. We found that the coding sequences of 5 S rDNA were highly conserved, including the A-, IE- and C-box regulatory elements (Figure S5). However, a base deletion was found in the NTS sequence of 5 S rDNA in the ROJ germplasm (Figure S6). In the ITS1 + 5.8 S + ITS2 sequence of 35 S rDNA, we found a variation of C and T bases at position + 2 bp of ITS2 (Figure S7). These results indicate that the sequences of these five accessions are highly conserved, with only a few base variations.

**Fig. 6:**
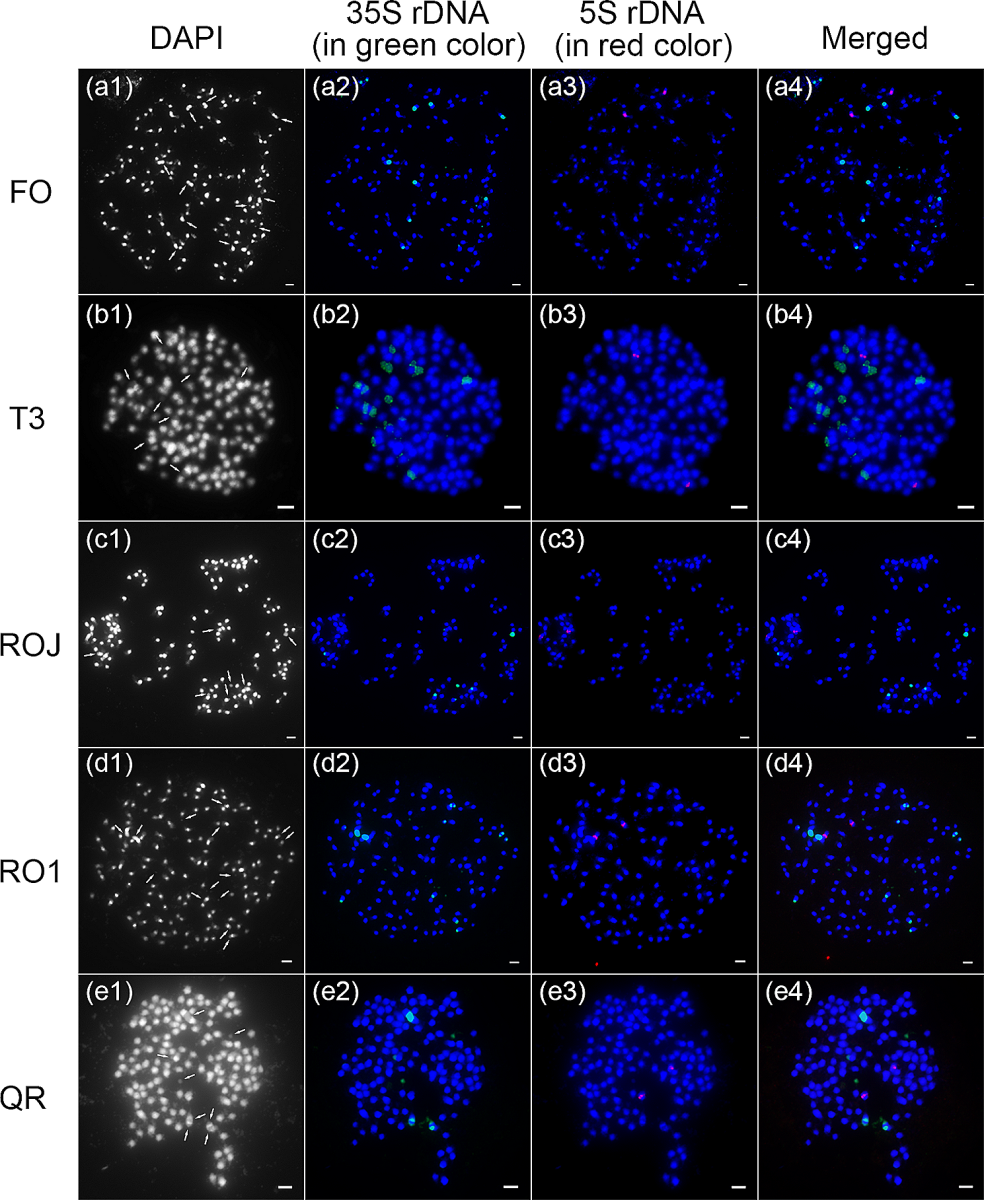
FISH localization of 5 S rDNA and 35 S rDNA families on mitotic metaphase chromosomes of five okra accessions. DAPI-stained mitotic chromosomes are shown in blue and grey. The signals of 35 S rDNA and 5 S rDNA are shown in green and red, respectively. The arrowheads point to the regions of relatively weak DAPI staining. (a1-a4) FISH mapping of 35 S rDNA and 5 S rDNA on the chromosomes of FO. (b1-b4) FISH mapping of 35 S rDNA and 5 S rDNA on the chromosomes of T3. (c1-c4) FISH mapping of 35 S rDNA and 5 S rDNA on the chromosomes of ROJ. (d1-d4) FISH mapping of 35 S rDNA and 5 S rDNA on the chromosomes of RO1. (e1-e4) FISH mapping of 35 S rDNA and 5 S rDNA on the chromosomes of QR. In the five okra accessions, we identified numerous chromosomes containing the 35 S rDNA loci, along with an additional pair of chromosomes containing the 5 S rDNA loci. Scale bars: 1 μm

By comparing the genomic proportions of 5 S rDNA and 35 S rDNA in NGS data, third-generation sequencing (TGS) data and assembled genomes, we found that all the data showed similar genomic proportions for 5 S rDNA, ranging from 0.13 to 0.49% (Figure S8). In addition, the NGS data showed similar genomic proportions for 35 S rDNA, ranging from 1.98 to 2.87% (Figure S8). However, the genomic proportion of assembled genomes for 35 S rDNA from the TGS and assembled genomes was much lower compared to the NGS data, suggesting 35 S rDNA is under-represented in the recently published okra genome assembly (Figure S8). To check the localization of 35 S and 5 S rDNA loci, we performed FISH analysis on metaphase chromosomes in five okra accessions using 35 S rDNA and 5 S rDNA probes. In all of the examined accessions, we detected multiple chromosomes containing the 35 S rDNA loci, as well as another chromosomal pair harboring the 5 S rDNA loci (Fig. 6). Interestingly, the intensity of 35 S rDNA hybridization signals varied across the chromosomes, and the signals were mainly located within regions of relatively weak DAPI staining (Fig. 6).

Unlike the 35S rDNA, the 5S rDNA loci seem to be less prone to homogenization in certain allopolyploids, graph clustering of Illumina reads corresponding to 5S rDNA could produce a simple circle in diploid species and more complex structures in allopolyploid genomes [30]. In the graphs, each vertex represents a sequence read and nodes connecting vertices depict sequence similarity between the reads. The loops were composed of vertices depicted in grey representing variable NTS regions in allopolyploid genomes [30]. Using this approach, we observed two loop structures interconnected by a junction region (annotated as the coding sequence of 5S rDNA) in five okra accessions, indicating the presence of their respective copies of 5S rDNA in the okra subgenome (Figure S9). Additionally, we observed varying numbers of grey vertices in the two loops, implying differences in their copy numbers within two subgenomes (Figure S9a-e). After assessing the copy number of 5S rDNA in both subgenomes of the okra variety ‘Wufu’ genome assembly, we observed that the copy number of 5 S rDNA in subgenome A (26,257) greatly exceeds that in subgenome B (546) (Figure S9f). Hence, in conjunction with the FISH results, we confirmed that 5 S rDNA predominantly localizes in subgenome A.

### Three satellite repeats with high GC content co-localized with 35 S rDNA

**Fig. 7:**
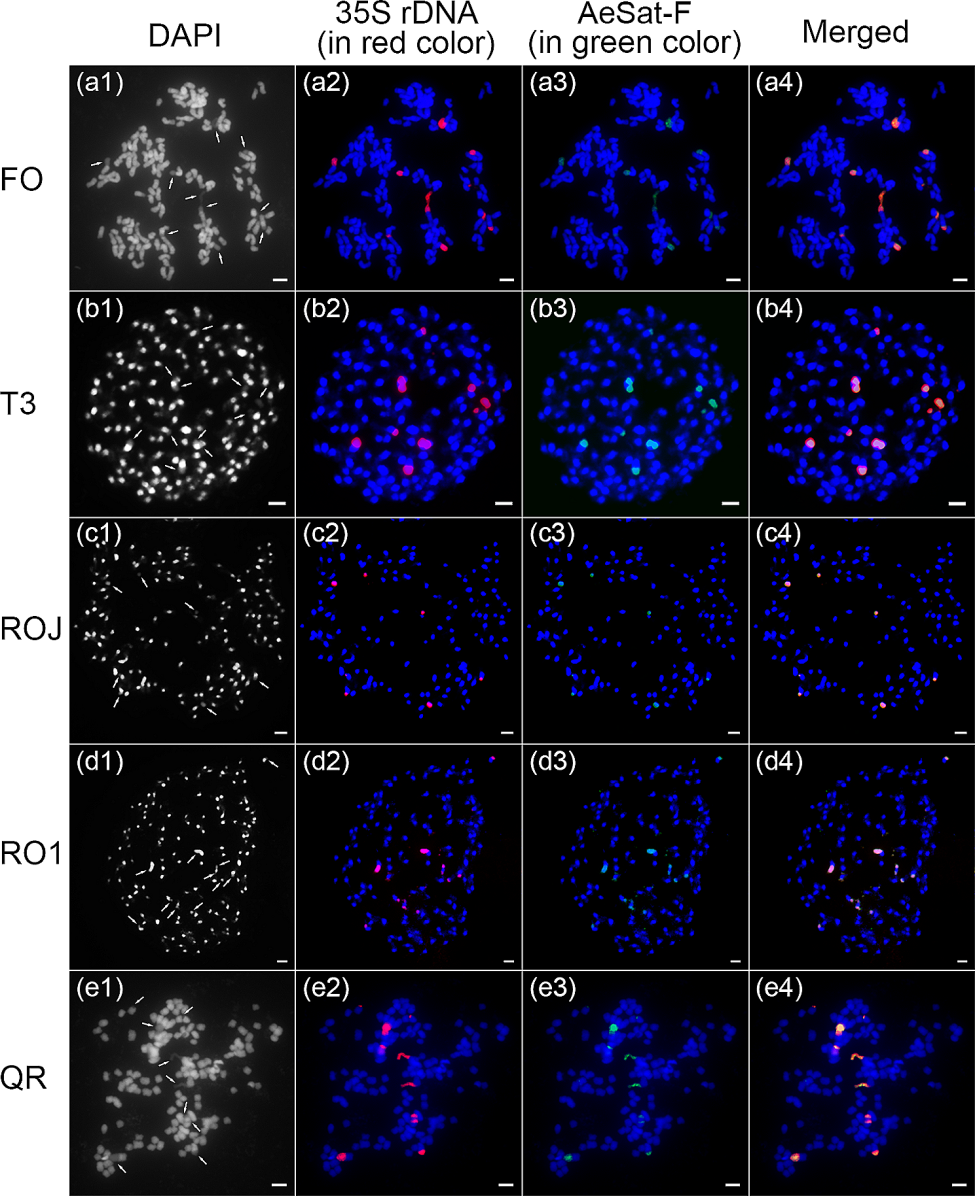
FISH mapping of 35 S rDNA and AeSat-F on the metaphase spreads of the five okra accessions. DAPI-stained mitotic chromosomes are shown in blue and grey. The signals of 35 S rDNA and AeSat-F are shown in red and green, respectively. The arrowheads point to the regions of relatively weak DAPI staining. (a1-a4) FISH mapping of 35 S rDNA and AeSat-F in FO. (b1-b4) FISH mapping of 35 S rDNA and AeSat-F in T3. (c1-c4) FISH mapping of 35 S rDNA and AeSat-F in ROJ. (d1-d4) FISH mapping of 35 S rDNA and AeSat-F in RO1. (e1-e4) FISH mapping of 35 S rDNA and AeSat-F in QR. AeSat-F exhibited a propensity for localizing in regions with relatively low DAPI staining, and it co-localized with the 35 S rDNA at the same chromosomal location. Scale bars: 1 μm

**Fig. 8:**
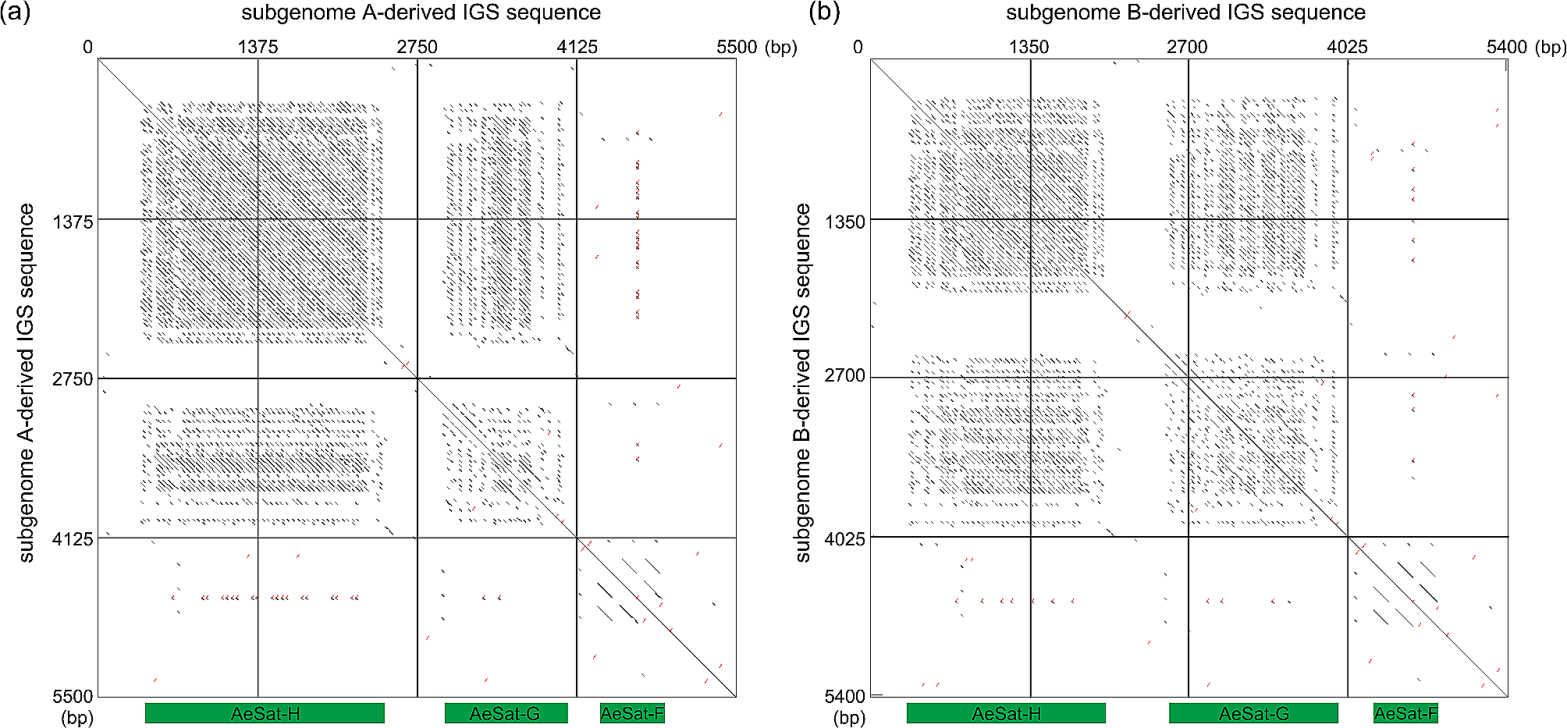
Dot-plot analysis of the IGS sequence of the 35 S rDNA from the assembled genome sequence of okra. Dot-plot representation illustrates the pairwise similarities between sub-repeats in the IGS sequence of the 35 S rDNA, indicating that three satellite repeats, namely AeSat-F, AeSat-G and AeSat-H, were derived from sub-repeats within the IGS sequence of subgenome A (**a**) and sB (**b**)

Based on the aforementioned FISH mapping experiments, we observed a consistent localization pattern for 35 S rDNA, AeSat-F, AeSat-G and AeSat-H. These sequences exhibited a preference for localizing within regions characterized by relatively weak DAPI staining. To validate the co-localization of these sequences, we conducted co-localization analyses using the 35 S rDNA probe in conjunction with the AeSat-F, AeSat-G, and AeSat-H satellite repeat probes on the same metaphase chromosome cells. FISH results demonstrated the co-localization of the satellite repeats from these three probes with 35 S rDNA at the same chromosomal locus (Fig. 7 and S10-11). Subsequently, we conducted sequence alignments of these three satellite repeats with the assembled genome sequence of okra. Remarkably, all three sequences were found to be situated within the IGS sequence of the 35 S rDNA (Fig. 8), and they all exhibited high sequence similarity with the IGS sequences of two okra subgenomes (average sequence similarity ranging from 86.8 to 97.8%). Specifically, AeSat-F and AeSat-H showed higher average sequence similarity with those of subgenome A, at 97.8% and 96.0% respectively, while AeSat-G exhibited higher average sequence similarity with those of subgenome B, at 91.1% (Table 2). Therefore, our findings confirmed that AeSat-F, AeSat-G and AeSat-H were satellite repeats derived from the IGS region of the 35 S rDNA.

**Table 2:** The average sequence similarity of the two subgenomic IGS sequences with AeSat-F, AeSat-G, and AeSat-H

	subgenome A-derived IGS	subgenome B-derived IGS
AeSat-F	97.8%	95.4%
AeSat-G	86.8%	91.1%
AeSat-H	96.0%	95.4%

## Discussion

In higher plants, centromeric sequences are generally composed of satellite repeats and centromeric retrotransposons (CRs), as demonstrated in sugarcane, rice, maize, and *B. distachyon* [19, 20, 23–29]. In our study, we discovered that five satellite repeats were consistently interspersed with the CRs lineage in okra (Fig. 4), indicating their association with centromeric satellite repeat identity in okra. Additionally, the abundant centromeric satellite repeats in higher plants form the most dominant tandem repeat families in the genomes. Similar characteristic has also been observed in okra, where five centromeric satellite repeats account for approximately 2.5% of the genome (Table 1). Intriguingly, in many higher plants, centromeres are typically characterized by the prevalence of a specific family of satellite repeats with considerable sequence diversity [31]. For instance, the centromeres in the model plant *A. thaliana* harbors several megabases of satellite repeat variants with a 79–89% sequence identity [32]. Similarly, we found that four out of these five centromeric satellite repeats in okra (AeSat-A, AeSat-B, AeSat-C and AeSat-D) exhibit a high degree of similarity (79.4–90.6%, Fig. 1 and S1). Moreover, the centromeric satellite repeat AeSat-E exhibits a low sequence similarity of 31.3–48.2% with these four centromeric satellite repeats, indicating that the centromeric satellite repeat sequences originate from two distinct families in okra (Fig. 1 and and S1). Satellite repeats undergo rapid changes during adaptation to new environments, which contribute to swift genome evolution and speciation [15–17]. In okra, there is a bias towards the predominance of centromeric repeats in a specific subgenome (Fig. 4). These findings provide novel insights into the sequence variation of centromeric repeats following polyploidization and asymmetrical evolution of centromeric repeats in allotetraploid okra subgenomes.

In most eukaryotes, centromeric satellite repeats are typically 150 to 180 bp in length, a length sufficient for wrapping around a single nucleosome [22, 33]. For example, CENP-A nucleosomes in *Homo sapiens* and CENH3 nucleosomes in *Oryza sativa* are highly phased on ~ 181 bp alpha satellite repeat and ~ 178 bp AthCEN178 satellite repeat, respectively [34, 35]. However, in some organisms like potato and chicken, centromeres can have multiple satellite arrays with varying unit sizes, including both repeat-containing and repeat-free sequences [24, 36]. In our study, our findings revealed four centromeric satellite repeats (AeSat-A, AeSat-B, AeSat-C and AeSat-D) have a near mono-nucleosomal length of 169–170 bp (Table 1). The remarkable consistency in length of the okra satellite repeats suggests the presence of a stringent length requirement. These observations suggest that these centromeric repeats may have evolved into a mature, centromere-adaptive structure in okra. However, one centromeric satellite repeat, AeSat-E, has a significantly different mono-nucleosomal length with 99 bp (Table 1). Similar to other plant species, satellite repeats with atypical monomeric lengths are likely unsuitable for centromeres [24].

Compared to the highly conserved coding sequences of 35 S rDNA and 5 S rDNA, the intergenic spacer sequences (ITS1 and ITS2 from 35 S rDNA, NTS from 5 S rDNA) exhibit more variability. Therefore, these intergenic spacer sequences serve as useful genetic markers for understanding interspecific and even intraspecific relationships [37, 38]. Traditionally, rDNA is thought to be evolved in a strict concerted evolution pattern, with little to no variation within species, but sequence diversity between species or higher taxa [39]. In the case of okra, there is limited intraspecific variability among five accessions, with only minor variations detected in the ITS2 and NTS region sequences (Figures S6 and S7). A cytogenetic study across 2,949 land plant species demonstrated that most species possess more 35 S rDNA loci than 5 S rDNA loci (47.79% of the investigated species). Conversely, for 19.05% of the species, the opposite pattern is observed; while the remaining 33.16% of species possess an equal number of loci [40]. Furthermore, there is a prevalent trend of an increase in the number of rDNA loci with ploidy level [40]. Nevertheless, in some species like Artemisia and Nicotiana, there is a potential decrease in the number of rDNA loci per monoploid genome following polyploidization [41–43]. This finding provides evidence for the occurrence of diploidization processes in polyploid plants over millions of years. In our study, we detected multiple 35 S rDNA loci (exceeding the expected ploidy level) and only two 5 S rDNA loci (less than the expected ploidy level) in five okra accessions using the FISH assay (Fig. 6), indicating a highly dynamic change in the number of rDNA loci during okra allopolyploidization. 35 S rDNA is fragile sites associated with epigenetic alterations and DNA damage under stress conditions, which could be one of the factors contributing to the significant variation in 35 S rDNA loci following polyploidization [44]. Furthermore, our examination of the clustering results of 5 S rDNA revealed a double ring configuration, implying the loss of 5 S rDNA from one okra subgenome after allopolyploidization (Figure S9). In chromosomes, the occurrence patterns of 5 S rDNA can be categorized into two types: the S-type arrangement, featuring long tandem repeats of regularly spaced units, and the L-type arrangement, characterized by solitary insertions are present within the intergenic spacer of the 35 S rDNA. The S-type arrangement is predominant in angiosperms, while the L-type arrangement is more characteristic of plant lineages that diverged early in angiosperm evolution and certain taxa within gymnosperms [40]. Our FISH assay revealed that 5 S rDNA are separated from 35 S rDNA in okra, thus suggesting its S-type arrangement, similar to most angiosperms.

In addition, we also found that three satellite DNAs in okra originated from the distinct IGS sub-repeats of 35 S rDNA (Fig. 8). Remarkably, several other plants, including potatoes, tomatoes, tobacco and pepper, were found to have highly amplified satellite DNAs that share sequence homology with the IGS sub-repeats of 35 S rDNA [42, 45–47]. However, in *Capsicum frutescen*, these satellite DNAs are even distributed in dispersed patterns across multiple chromosomes [42], whereas this pattern has not been observed in okra (Fig. 7). Furthermore, in both the 35 S rDNA and IGS regions of okra, we observed that they were predominantly located in DAPI-negative staining regions, suggesting the presence of GC-rich heterochromatin and the scarcity of AT-rich DNA in these regions (Figs. 3, 6 and 7, S10 and S11). A similar phenomenon has been documented in other species, such as *Rhodeus amarus* [48].

TGS technologies greatly improve the accuracy of genome assemblies, as they are capable of accurately reading and capturing complete DNA fragments of 10 kilobases or longer. This allows for the coverage of satellite repeats [18]. However, the strategies employed by these technologies are applicable to shorter arrays of less complex repeats that traverse several kilobases. In the case of okra, we identified arrays of the centromeric satellite repeats (AeSat-A, AeSat-B, AeSat-C, AeSat-D and AeSat-E) and 5 S rDNA through FISH analysis (Figs. 2 and 6), which were accurately assembled on the okra genome assembly. Nevertheless, accurately assembling genomes is still a challenge due to the presence of complex repetitive regions, such as arrays that span hundreds to thousands of kilobases and are comprised of units that are several kilobases in length, including 35 S rDNA. For instance, the recently published near T2T genome of *A. thaliana* still contains inaccuracies specifically related to highly repeated DNA sequences including the short-arm 35 S rDNA clusters and adjacent telomeres on chromosomes 2 and 4 [20]. In our study, we also noticed that they are under-represented in the recently published okra genome assembly (Figure S8), possibly due to the aforementioned challenges. Additionally, the presence of three complex satellite sequences on the IGS, along with their highly GC-rich content, further exacerbates the difficulty in its genome assembly.

## Conclusion

In this study, we examined the satellite repeat patterns in five okra accessions. We used both genomic and cytogenetic methods to identify and analyze these satellite repeats. We discovered that each of the five accessions had eight distinct satellite repeats, which showed a significant degree of similarity among these accessions. By conducting FISH experiments, we observed that these satellite repeats were present in multiple loci and had variations in their distribution across the chromosomes. Interestingly, we found that five of these satellite repeats were interspersed with retrotransposons located near the centromeres, indicating their potential involvement in defining the identity of satellite repeats in the centromeric region. Through the analysis of existing genome assemblies and dual-color FISH, we confirmed that the amplification patterns of these satellite repeats were biased towards specific subgenomes, suggesting distinct evolutionary dynamics within the allopolyploid subgenome. Additionally, using FISH assay, we observed the presence of multiple chromosomes containing the 35 S rDNA loci, as well as another pair of chromosomes carrying the 5 S rDNA loci in okra. Notably, the intensity of the 35 S rDNA hybridization signals varied across the chromosomes, with all signals localized within regions of relatively weak DAPI staining, which are associated with GC-rich heterochromatin. Lastly, we discovered a similar localization pattern between the 35 S rDNA and three satellite repeats possessing a high GC content, confirming their origin in the intergenic spacer region of the 35 S rDNA. Overall, our study provides new insights into the unique satellite repeat patterns in okra, shedding light on their composition, distribution, and evolutionary dynamics in complex allopolyploid genomes.

## Methods

### Plant material and genomic DNA isolation

Five okra accessions, including Fruit Okra (FO), Titanic No.3 (T3), Red Okra JPN (ROJ), Red Okra No.1 (RO1) and QK-RP-2-1 (QR), were used in this study. The phenotypic traits, such as fruit color, plant height, fruit number, and fruit shape, of these five okra accessions from diverse geographical regions manifest substantial variations. In our okra hybrid breeding program, the successful production of the F_1_ generation through the crossbreeding of five phenotypically distinct accessions indicated their potential closely related genetic relationships. Consequently, we aim to conduct satellite sequence analysis to investigate the evolutionary patterns of satellite sequences in these five allopolyploid okra genomes. These accessions were cultivated in a glasshouse located at Minjiang University in Fujian, China. To obtain DNA samples, fresh leaves were collected from each of the five accessions and subjected to DNA extraction using the CTAB method.

### Genome-wide identification of repetitive DNA

Identification of repetitive DNA was achieved using a graph-based sequence similarity clustering analysis from randomly selected 2 M pair-end reads for each accession as described by Novak et al. [21]. Initially, the genomic DNA from the five accessions was subjected to low coverage sequencing using the Illumina HiSeq platform. Quality assessment and filtering of low-quality reads were performed using FastQC and Trimmomatic v0.36. To conduct graph-based clustering analysis, a set of two million randomly selected reads was analyzed using the Galaxy RepeatExplorer2 web platform (https://repeatexplorer-elixir.cerit-sc.cz/galaxy/). Reads with graph nodes showing similar distribution patterns were categorized into the same repeat sequence families. For subsequent analyses, the representative sequences from each satellite sequence family were extracted based on the presence of tandem sub-repeats within their read or assembled contig sequences.

### Sequence analysis and alignment of 5 S rDNA and 35 S rDNA

Using the TAREAN tool in the Galaxy RepeatExplorer2 web platform (https://repeatexplorer-elixir.cerit-sc.cz/galaxy/), repeat sequence clusters of 5 S rDNA (5 S rDNA + NTS) and 35 S rDNA (ITS1 + 5.8 S + ITS2) were extracted from the NGS data of five okra varieties. From these two clusters, representative contigs were selected for sequence analysis and alignment. Finally, sequence analysis and alignment of 5 S rDNA (5 S rDNA + NTS) and 35 S rDNA (ITS1 + 5.8 S + ITS2) from the five okra varieties were performed using the default parameters of the DNAMAN 8 software.

### PCR amplification and probe preparation

PCR amplification was conducted using fusion PCR for the full-length satellite repeats (Table S2). PCR amplification was performed in a 20-µL volume containing 100 ng genomic DNA, 200 µM dNTPs, 1× ExTaq Buffer, 250 nM primers, and 1 U ExTaq DNA polymerase (TaKaRa Bio, Kusatsu, Shiga, Japan). The PCR conditions were as follows: an initial denaturation at 95 °C for 3 min, followed by 35 amplification cycles of denaturation at 95 °C for 30 s, annealing at 55 °C for 30 s, extension at 72 °C for 30 s, and a final extension at 72 °C for 10 min. Following PCR amplification, the products were analyzed on a 1% (w/v) agarose gel electrophoresis. Moreover, the resulting PCR products were labeled using nick translation with either biotin dUTP or digoxigenin dUTP (Roche, http://www.roche.com).

### Chromosome preparation and FISH

Chromosome preparation and FISH were conducted following the previously described methods [25]. Root tips were pretreated with an 8-hydroxyquinoline solution at room temperature for 2 h before fixation in 3:1 ethanol: glacial acetic acid for 24 h. Subsequently, the root tips were treated with an enzyme mixture (1% pectolyase Y23, 2% pectolyase, 2% cellulase RS, and 4% Onozuka R-10 cellulase) at 37 °C for 3 h. The resulting cell suspension was dropped onto slides. Denatured probes were mixed with a hybridization mixture (50% formamide and 20% dextran sulfate in 2× SSC) and applied to denatured chromosome on slides. A cover slip was added to cover the mixture, and the slides were incubated overnight in a hybridization chamber at 37 °C. Following incubation, the slides were washed with 2× SSC three times for 5 min each and 1× PBS for 5 min at room temperature. The labeled probes were detection using either streptavidin-conjugated Alexa Fluor 488 (Invitrogen, http://www.invitrogen.com) or rhodamine-conjugated anti-digoxigenin antibodies (Roche, https://www.sigmaaldrich.cn/CN/zh/product/roche). Finally, the slides were counterstained with 4’,6-diamidino-2-phenylindole (DAPI), and images were captured using an Olympus BX63 fluorescence microscope equipped with an Olympus DP80 CCD camera (Olympus, https://www.getolympus.com). The captured images were processed using cellsens dimension 1.9. The contrast of the images was adjusted using photoshop CC (Adobe, https://www.adobe.com).

### Electronic supplementary material

Below is the link to the electronic supplementary material.

**Table S1**. The origin of the five okra accessions. **Table S2**. The sequences of the full-length satellite repeats. **Figure S1**. Multiple sequence alignment of eight satellite repeats in five okra accessions. **Figure S2**. FISH localization of five centromere satellite repeats on mitotic metaphase chromosomes of FO accession. (a) Dual-color FISH of AeSat-B (red) and AeSat-E (green), showing colocalization of these two satellite repeats on the chromosomes of FO; (b) Dual-color FISH of AeSat-B (red) and AeSat-A (green), showing non-colocalization of these two satellite repeats on the chromosomes of FO; (c) Dual-color FISH of AeSat-B (red) and AeSat-C (green), showing non-colocalization of these two satellite repeats except on one pair of chromosomes of FO; (d) Dual-color FISH of AeSat-B (red) and AeSat-D (green), showing colocalization of these two satellite repeats on a few chromosomes of FO. Thus, five centromeric satellite repeats exhibit distribution bias within the two subgenomes in FO accession. Scale bars: 1 μm. **Figure S3**. FISH localization of five centromere satellite repeats on mitotic metaphase chromosomes of T3 accession. (a) Dual-color FISH of AeSat-B (red) and AeSat-E (green), showing colocalization of these two satellite repeats on the chromosomes of T3; (b) Dual-color FISH of AeSat-B (red) and AeSat-A (green), showing non-colocalization of these two satellite repeats on the chromosomes of T3; (c) Dual-color FISH of AeSat-B (red) and AeSat-C (green), showing non-colocalization of these two satellite repeats except on one pair of chromosomes of T3; (d) Dual-color FISH of AeSat-B (red) and AeSat-D (green), showing colocalization of these two satellite repeats on a few chromosomes of T3. Thus, five centromeric satellite repeats exhibit distribution bias within the two subgenomes in T3 accession. Scale bars: 1 μm. **Figure S4**. FISH localization of five centromere satellite repeats on mitotic metaphase chromosomes of ROJ accession. (a) Dual-color FISH of AeSat-B (red) and AeSat-E (green), showing colocalization of these two satellite repeats on the chromosomes of ROJ; (b) Dual-color FISH of AeSat-B (red) and AeSat-A (green), showing non-colocalization of these two satellite repeats on the chromosomes of ROJ; (c) Dual-color FISH of AeSat-B (red) and AeSat-C (green), showing non-colocalization of these two satellite repeats except on one pair of chromosomes of ROJ; (d) Dual-color FISH of AeSat-B (red) and AeSat-D (green), showing colocalization of these two satellite repeats on a few chromosomes of ROJ. Thus, five centromeric satellite repeats exhibit distribution bias within the two subgenomes in ROJ accession. Scale bars: 1 μm. **Figure S5**. FISH localization of five centromere satellite repeats on mitotic metaphase chromosomes of QR accession. (a) Dual-color FISH of AeSat-B (red) and AeSat-E (green), showing colocalization of these two satellite repeats on the chromosomes of QR; (b) Dual-color FISH of AeSat-B (red) and AeSat-A (green), showing non-colocalization of these two satellite repeats on the chromosomes of QR; (c) Dual-color FISH of AeSat-B (red) and AeSat-C (green), showing non-colocalization of these two satellite repeats except on one pair of chromosomes of QR; (d) Dual-color FISH of AeSat-B (red) and AeSat-D (green), showing colocalization of these two satellite repeats on a few chromosomes of QR. Thus, five centromeric satellite repeats exhibit distribution bias within the two subgenomes in QR accession. Scale bars: 1 μm. **Figure S6**. Multiple sequence alignment of 5S rDNA and NTS sequence from five okra accessions. Regions of the 5S rDNA and NTS sequence are shaded in the grey and blue box, respectively. The A-, IE- and C-Boxes of pol III promoters are marked in green. *Figure S7*. Multiple sequence alignment of ITS1+5.8S+ITS2 sequence from five okra accessions. Regions of the 5.8S rDNA as well as ITS1 and ITS2 sequence are shaded in the grey and blue box, respectively. **Figure S8**. The genomic proportions of 35S rDNA and 5S rDNA in NGS, TGS and genome assembly. **Figure S9**. The 5S rDNA sequence reads organized in graph structures from the RepeatExplorer2 graphical output. (a-e) Single reads are represented by vertices (nodes) and their sequence overlaps by edges. The 5S rDNA coding sequences and intergenic spacers are highlighted in green and grey vertices, respectively. Two loop structures were interconnected by a junction region (annotated as the coding sequence of 5S rDNA) in five okra accessions. Arrows indicate two intergenic spacers of 5S rDNA. (f) The copy number of 5S rDNA in both subgenomes of the okra variety ‘Wufu’ genome assembly. **Figure S10**. FISH mapping of 35S rDNA and AeSat-G on the metaphase spreads of the five okra accessions. DAPI-stained mitotic chromosomes are shown in blue and grey. The signals of 35S rDNA and AeSat-G are shown in red and green, respectively. (a1-a4) FISH mapping of 35S rDNA and AeSat-G in FO. (b1-b4) FISH mapping of 35S rDNA and AeSat-G in T3. (c1-c4) FISH mapping of 35S rDNA and AeSat-G in ROJ. (d1-d4) FISH mapping of 35S rDNA and AeSat-G in RO1. (e1-e4) FISH mapping of 35S rDNA and AeSat-G in QR. AeSat-G exhibited a propensity for localizing in regions with relatively low DAPI staining, and it co-localized with the 35S rDNA at the same chromosomal location. Scale bars: 1 μm. **Figure S11**. FISH mapping of 35S rDNA and AeSat-H on the metaphase spreads of the five okra accessions. DAPI-stained mitotic chromosomes are shown in blue and grey. The signals of 35S rDNA and AeSat-H are shown in red and green, respectively. (a1-a4) FISH mapping of 35S rDNA and AeSat-H in FO. (b1-b4) FISH mapping of 35S rDNA and AeSat-H in T3. (c1-c4) FISH mapping of 35S rDNA and AeSat-H in ROJ. (d1-d4) FISH mapping of 35S rDNA and AeSat-H in RO1. (e1-e4) FISH mapping of 35S rDNA and AeSat-H in QR. AeSat-H exhibited a propensity for localizing in regions with relatively low DAPI staining, and it co-localized with the 35S rDNA at the same chromosomal location. Scale bars: 1 μm.

## Data Availability

The datasets necessary for supporting the results of this article are included in this manuscript and its additional files. The datasets generated and/or analyzed during the current study are available in the National Center for Biotechnology Information (NCBI) repository with accession number BioProject PRJNA1005429.
