# Supplementary material for: Genomic and cytogenetic analyses reveal satellite repeat signature in allotetraploid okra (Abelmoschus esculentus)
Source: BMC Plant Biol. 2024 Jan 25;24:71. doi: 10.1186/s12870-024-04739-9 (PMC10809672; doi:10.1186/s12870-024-04739-9)
Supplement: Supplementary file 1 — Table S1. The origin of the five okra accessions. Table S2. The sequences of the full-length satellite repeats. Figure S1. Multiple sequence alignment of eight satellite repeats in five okra accessions. Figure S2. FISH localization of five centromere satellite repeats on mitotic metaphase chromosomes of FO accession. (a) Dual-color FISH of AeSat-B (red) and AeSat-E (green), showing colocalization of these two satellite repeats on the chromosomes of FO; (b) Dual-color FISH of AeSat-B (red) and AeSat-A (green), showing non-colocalization of these two satellite repeats on the chromosomes of FO; (c) Dual-color FISH of AeSat-B (red) and AeSat-C (green), showing non-colocalization of these two satellite repeats except on one pair of chromosomes of FO; (d) Dual-color FISH of AeSat-B (red) and AeSat-D (green), showing colocalization of these two satellite repeats on a few chromosomes of FO. Thus, five centromeric satellite repeats exhibit distribution bias within the two subgenomes in FO accession. Scale bars: 1 μm. Figure S3. FISH localization of five centromere satellite repeats on mitotic metaphase chromosomes of T3 accession. (a) Dual-color FISH of AeSat-B (red) and AeSat-E (green), showing colocalization of these two satellite repeats on the chromosomes of T3; (b) Dual-color FISH of AeSat-B (red) and AeSat-A (green), showing non-colocalization of these two satellite repeats on the chromosomes of T3; (c) Dual-color FISH of AeSat-B (red) and AeSat-C (green), showing non-colocalization of these two satellite repeats except on one pair of chromosomes of T3; (d) Dual-color FISH of AeSat-B (red) and AeSat-D (green), showing colocalization of these two satellite repeats on a few chromosomes of T3. Thus, five centromeric satellite repeats exhibit distribution bias within the two subgenomes in T3 accession. Scale bars: 1 μm. Figure S4. FISH localization of five centromere satellite repeats on mitotic metaphase chromosomes of ROJ accession. (a) Dual-color FISH of AeSat-B (red) a [file 12870_2024_4739_MOESM1_ESM.doc]

Table S1. The origin of the five okra accessions

| No.1 | Accession name | Origin | Latitude | Longitude |
| --- | --- | --- | --- | --- |
| 1 | Fruit Okra (FO) | Shijiazhuang, Hebei, China | 38°03′ N | 114°29′ E |
| 2 | Titanic No.3 (T3) | Mianyang, Sichuan, China | 31°29′ N | 104°44′ E |
| 3 | Red Okra JPN (ROJ) | Tokyo, Japan | 35°41′ N | 139°44′ E |
| 4 | Red Okra No 1 (RO1) | Nagoya, Japan | 35°11′ N | 136°56′ E |
| 5 | QK-RP-2-1 (QR) | Haikou, Hainan, China | 110°10′ N | 20°03′ E |

Table S2. The sequences of the full-length satellite repeats

| Name | Sequence (5'→3') |
| --- | --- |
| AeSat-A-FO | ATCATTTTCGATCGTTTTTTAGGGTTTAGAGCGTGCATGTCTAAATGGGCTTTATAGTGAACCCGTTCGAATTTTAACCGAGTAAAAAGCTCAAAATTTGAGTTTAAGCTGTATAATTAGGTTCAAAAGCCCATAATTAACTATTTGAACCCATAAAATTTGATTTAAT |
| AeSat-A-T3 | ATCATTTTCGATCGTTTTTTAGGGTTTAGAGCGTGCATGTCTAAATGGGCTTTATAGTGAACCCGTTCGAATTTTAACCGAGTAAAAAGCTCAAAATTTGAGTTTAAGCTGTATAATTAGGTTCAAAAGCCCATAATTAACTATTTGAACCCATAAAATTTGATTTAAT |
| AeSat-A-ROJ | ATCATTTTCGATCGTTTTTTAGGGTTTAGAGCGTGCATGTCTAAATGGGCTTTATAGTGAACCCGTTCGAATTTTAACCGAGTAAAAAGCTCAAAATTTGAGTTTAAGCTGTATAATTAGGTTCAAAAGCCCATAATTAACTTTTTGAACCCATAAAATTTGATTTAAT |
| AeSat-A-RO1 | ATCATTTTCGATCGTTTTTTAGGGTTTAGAGCGTGCATGTCTAAATGGGCTTTATAGTGAACCCGTTCGAATTTTAACCGAGTAAAAAGCTCAAAATTTGAGTTTAAGCTGTATAATTAGGTTCAAAAGCCCATAATTAACTTTTTGAACCCATAAAATTTGATTTAAT |
| AeSat-A-QR | ATCATTTTCGATCGTTTTTTAGGGTTTAGAGCGTGCATGTCTAAATGGGCTTTATAGTGAACCCGTTCGAATTTTAACCGAGTAAAAAGCTCAAAATTTGAGTTTAAGCTGTATAATTAGGTTCAAAAGCCCATAATTAACTTTTTGAACCCATAAAATTTGATTTAAT |
| AeSat-B-FO | ACATTTGGTTGTCGTTTTGTTGGGATTAAAACGTGCATGTCTAAATGGGCTTTATAGAGAATCCGTTCGAATTTTAACCGAGTAAAAAGCTGAAAATTTTAGATTAATCTGTATAATTTTGTTCAAAAGCCCATAATTAACTATTTGAACTCATAAAATTTGATTTAATA |
| AeSat-B-T3 | ACATTTGGTTGTCGTTTTGTTGGGATTAAAACGTGCATGTCTAAATGGGCTTTATAGAGAATCCGTTCGAATTTTAACCGAGTAAAAAGCTGAAAATTTTAGATTAATCTGTATAATTTTGTTCAAAAGCCCATAATTAACTATTTGAACTCATAAAATTTGATTTAATA |
| AeSat-B-ROJ | ACATTTGGTTGTCGTTTTGTTGGGATTAAAACGTGCATGTCTAAATGGGCTTTATAGAGAATCCGTTCGAATTTTAACCGAGTAAAAAGCTGAAAATTTGAGATTAATCTGTATAATTTTGTTCAAAAGCCCATAATTAACTATTTGAACTCATAAAATTTGATTTAATA |
| AeSat-B-RO1 | ACATTTGGTTGTCGTTTTGTTGGGATTAAAACGTGCATGTCTAAATGGGCTTTATAGAGAATCCGTTCGAATTTTAACCGAGTAAAAAGCTGAAAATTTTAGATTAATCTGTATAATTTTGTTCAAAAGCCCATAATTAACTATTTGAACTCATAAAATTTGATTTAATA |
| AeSat-B-QR | ACATTTGGTTGTCGTTTTGTTGGGATTAAAACGTGCATGTCTAAATGGGCTTTATAGAGAATCCGTTCGAATTTTAACCGAGTAAAAAGCTGAAAATTTTAGATTAATCTGTATAATTTTGTTCAAAAGCCCATAATTAACTATTTGAACTCATAAAATTTGATTTAATA |
| AeSat-C-FO | TCTCATTTTGTGACCGTTTTGTTGGGTTTAAAGCGTGCATTTCTAATTGGGCTTTACAGTAAAACAGTTGAAATTTTAACTGAGTAAAATACTCTAAAATTGAAATTAAGCTGTATAATCAGATTCAAAAGCCTATAATTAAATATTTGAACTCATAAAATTTGAATTAA |
| AeSat-C-T3 | TCTCATTTTGTGACCGTTTTGTTGGGTTTAAAGCGTGCATTTCTGATTGGGCTTTACAGTAAAACAGTTGAAATTTTAACTGAGTAAAATACTCTAAAATTGAAATTAAGCTGTATAATCAGATTCAGAAGCCTATAATTAAATATTTGAACTCATAAAATTTGAATTAA |
| AeSat-C-ROJ | TCTCATTTTGTGACCGTTTTGTTGGGTTTAAAGCGTGCATTTCTAATTGGGCTTTACAGTAAAACAGTTGAAATTTTAACTGAGTAAAATACTCTAAAATTGAGATTAAGCTGTATAATCAGATTCAAAAGCCTATAATTAAATATTTGAACTCATAAAATTTGAATTAA |
| AeSat-C-RO1 | TCTCATTTTGTGACCGTTTTGTTGGGTTTAAAGCGTGCATTTCTAATTGGGCTTTACAGTAAAACCGTTGAAATTTTAACTGAGTAAAATACTCTAAAATTGAAATTAAACTGTATAATCAGATTCAAAAGCCTATAATTAAATATTTGAACTCATAAAATTTGAATTAA |
| AeSat-C-QR | TCTCATTTTGTGACCGTTTTGTTGGGTTTAAAGCGTGCATTTCTAATTGGGCTTTACAGTAAAACCGTTGAAATTTTAACTGAGTAAAATACTCTAAAATTGAAATTAAGCTGTATAATCAGATTCAAAAGCCTATAATTAAATATTTGAACTCATAAAATTTGAATTAA |
| AeSat-D-FO | TCATTTTGTGGCCGTTTTGTTAGGTTTAAAGTGTGCATATCTAAATGAGCTTTATAGTGAACACATTGGAATTTAAACCGAGTAAAATGCTCTAAAATTGAGATTAAGTGGTTTAATCATATTCAAAAGCCCATAATTAACTATTTGAACTCATGAAATTTGATTTAATC |
| AeSat-D-T3 | TTATTTTGTGGCCGTTTTGTTAGGTTTAAAGTGTGCATGTCTAATTGAGCTTTATAGTGAACACATTGGAATTTAAACCGAGTAAAATGCTCTAAAATTGAGATTAAGTTGTTTAATCATATTCAAAAGCCCATAATTAACTATTTGAACTCATGAAATTTGATTTATTC |
| AeSat-D-ROJ | TTATTTTGTGGCCGTTTTGTTAGGTTTAAAGTGTGCATGTCTAATTGAGCTTTATAGTGAACACATTGGAATTTAAACCGAGTAAAATGCTCTAAAATTGAGATTAAGTTGTTTAATCATATTCAAAAGCCCATAATTAACTATTTGAACTCATGAAATTTGATTTATTC |
| AeSat-D-RO1 | TTATTTTGTGGCCGTTTTGTTAGGTTTAAAGTGTGCATATCTAATTGAGCTTTTTAGTGAACACATTGGAATTTAAACCGAGTAAAATGCTCTAAAATTGAGATTAAGTTGTTTAATCATATTCAAAAGCCCATAATTAACTATTTGAACTCATGAAATTTGATTTAATC |
| AeSat-D-QR | TTATTTTGTGGCCGTTTTGTTAGGTTTAAAGTGTGCATGTCTAATTGAGCTTTATAGTGAACACATTGGAATTTAAACCGAGTAAAATGCTCTAAAATTGAGATTAAGTTGTTTAATCATATTCAAAAGCCCATAATTAACTATTTGAACTCATGAAATTTGATTTATTC |
| AeSat-E-FO | AGAGAGGGGTCATTCATCACGACATACGCTCAAAGAGAGGGGTCATTCATCACGACATACGCTCAAAGAGAGGGGTCATTCATCACGACATACGCTCAA |
| AeSat-E-T3 | ACGGTATACGCTCAAAGAGAGGGACCGTTCATTATGACATGCACTCGAAGAGAGGGGTCATTCATCGCGACATACGCTCAAAGAGAGGGGTCGTCCATC |
| AeSat-E-ROJ | ACGGTATACGCTCAAAGAGAGGGACCGTTCATTATGACATGCACTCGAAGAGAGGGGTCATTCATCGCGACATACGCTCAAAGAGAGGGGTCGTCCATC |
| AeSat-E-RO1 | AGAGAGGGGTCATTCATCACGACATACGCTCAAAGAGAGGGGTCATTCATCACGACATACGCTCAAAGAGAGGGGTCATTCATCACGACATACGCTCAA |
| AeSat-E-QR | ACGGTATACGCTCAAAGAGAGGGACCGTTCATTATGACATGCACTCGAAGAGAGGGGTCATTCATCGCGACATACGCTCAAAGAGAGGGGTCGTCCATC |
| AeSat-F-FO | TTGGTTTTGTCTCCGTGTGATCTTGTTGGCTCTGTGCTACTTGCCGAGACCCCGTCACCCCGGTACCAAGTCCCTTTGAGGCAACGGCCCACTGGGGGGTGCGAGTAGACCATCTAATGTTCATGCATCTGAAAGGACCGAGGCCTCGGGGTGTTTCCCTTTCCGGGTCTCGTCAAACACGATTTTGGAAAATCTCGTGGAT |
| AeSat-F-T3 | TCGGTTTTTTCTCCGTGTGATCTTGTTGGCTCTGTGCTACTTGCCGAGACCCCGTCACCCCGGTACCAAGTCCCTTTGAGGCAACGGCCCACTGGGGGGTGCGAGTAGACCATCTAATGTTCATGCATCTGATAGGACCGAGGCCTCGGGGTGTTTCCCTTTCCGGGTCTCGTCAAACACGATTTTGGAAAATCTCGTGGAT |
| AeSat-F-ROJ | TCGGTTTTTTCTCCGTGTGATCTTGTTGGCTCTGTGCTACTTGCCGAGACCCCGTCACCCCGGTACCAAGTCCCTTTGAGGCAACGGCCCACTGGGGGGTGCGAGTAGACCATCTAATGTTCATGCATCTGAAAGGACCGAGGCCTCGGGGTGTTTCCCTTTCCGGGTCTCGTCAAACACGATTTTGGAAAATCTCGTGGAT |
| AeSat-F-RO1 | TCGGTTTTTTCTCCGTGTGATCTTGTTGGCTCTGTGCTACTTGCCGAGACCCCGTCACCCCGGTACCAAGTCCCTTTGAGGCAACGGCCCACTGGGGGGTGCGAGTAGACCATCTAATGTTCATGCATCTGAAAGGACCGAGGCCTCGGGGTGTTTCCCTTTCCGGGTCTCGTCAAACACGATTTTGGAAAATCTCGTGGAT |
| AeSat-F-QR | TCGGTTTTTTCTCCGTGTGATCTTGTTGGCTCTGTGCTACTTGCCGAGACCCCGTCACCCCGGTACCAAGTCCCTTTGAGGCAACGGCCCACTGGGGGGTGCGAGTAGACCATCTAATGTTCATGCATTCGAAAGGACCGAGGCCTCGGGGTGTTTCCCTTTCCGGGTCTCGTCAAACACGATTTTGGAAAATCTCGTGGAT |
| AeSat-G-FO | CGTCCGCACCCATGTGCCGACTTCCCCCGATGGTCACCGGGTGTCTCGCACCCATGGTCGGGGTTCACCCCGATGGTGTCCGAGGCTGCCGCACCCCAGGGGCTCTTTCTCCGCGATGGTGACCGAGTACCTCGCA |
| AeSat-G-T3 | CGTCCGCACCCATGTGCCGACTTCCCCCGATGGTCACCGGGTGTCTCGCACCCATGGTCGGGGTTCACCCCGATGGTGTCCGAGGCTGCCGCACCCCAGGGGCTCTTTCTCCGCGATGGTGACCGAGTACCTCGCA |
| AeSat-G-ROJ | CGTCCGCACCCATGTGCCGACTTCCCCCGATGGTCACCGGGTGTCTCGCACCCATGGTCGGGGTTCACCCCGATGGTGTCCGAGGCTGCCGCACCCCAGGGGCTCTTTCTCCGCGATGGTGACCGAGTACCTCGCA |
| AeSat-G-RO1 | CGTCCGCACCCATGTGCCGACTTCCCCCGATGGTCACCGGGTGTCTCGCACCCATGGTCGGGGTTCACCCCGATGGTGTCCGAGGCTGCCGCACCCCAGGGGCTCTTTCTCCGCGATGGTGACCGAGTACCTCGCA |
| AeSat-G-QR | CGTCCGCACCCATGTGCCGACTTCCCCCGATGGTCACCGGGTGTCTCGCACCCATGGTCGGGGTTCACCCCGATGGTGTCCGAGGCTGCCGCACCCCAGGGGCTCTTTCTCCGCGATGGTGACCGAGTACCTCGCA |
| AeSat-H-FO | GAACCCGGACCATGGGTGCGGAAGCCTCGGTCACCATCGGGGT |
| AeSat-H-T3 | GAACCCGGACCATGGGTGCGGAAGCCTCGGTCACCATCGGGGT |
| AeSat-H-ROJ | GAACCCGGACCATGGGTGCGGAAGCCTCGGTCACCATCGGGGT |
| AeSat-H-RO1 | GAACCCGGACCATGGGTGCGGAAGCCTCGGTCACCATCGGGGT |
| AeSat-H-QR | GAACCCGGACCATGGGTGCGGAAGCCTCGGTCACCATCGGGGT |


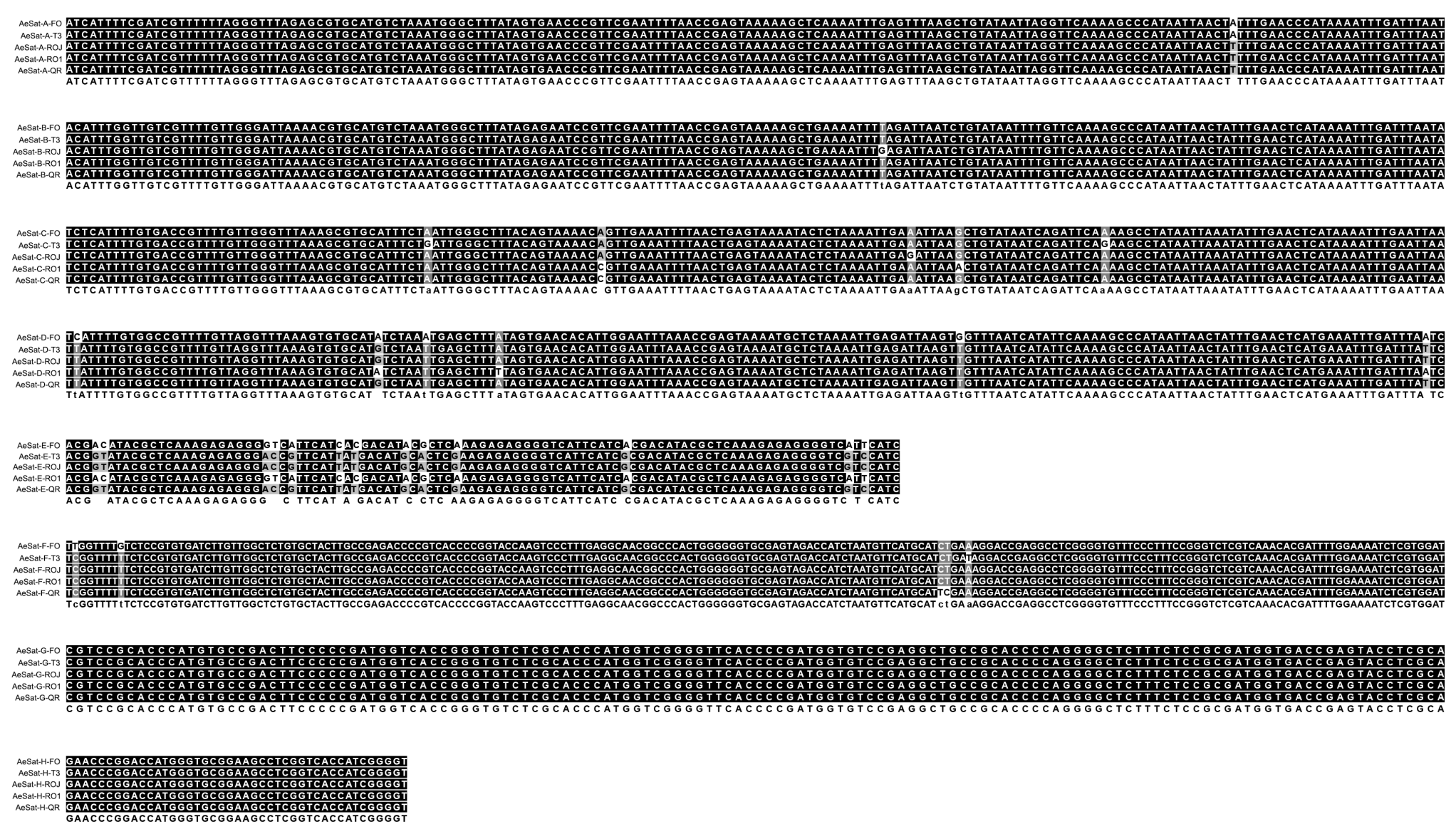


Figure S1. Multiple sequence alignment of eight satellite repeats in five okra accessions.


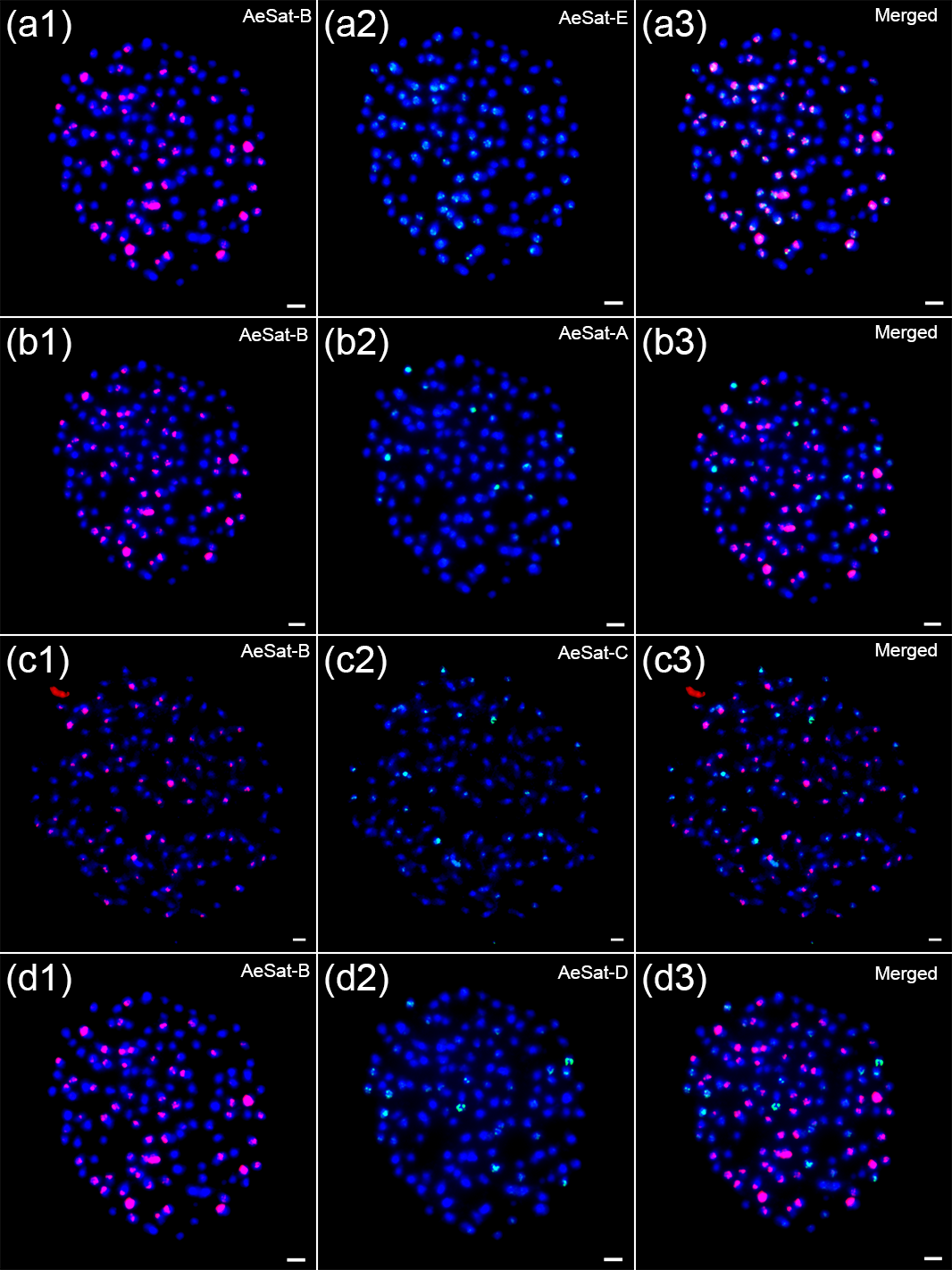


Figure S2. FISH localization of five centromere satellite repeats on mitotic metaphase chromosomes of FO accession. (a) Dual-color FISH of AeSat-B (red) and AeSat-E (green), showing colocalization of these two satellite repeats on the chromosomes of FO; (b) Dual-color FISH of AeSat-B (red) and AeSat-A (green), showing non-colocalization of these two satellite repeats on the chromosomes of FO; (c) Dual-color FISH of AeSat-B (red) and AeSat-C (green), showing non-colocalization of these two satellite repeats except on one pair of chromosomes of FO; (d) Dual-color FISH of AeSat-B (red) and AeSat-D (green), showing colocalization of these two satellite repeats on a few chromosomes of FO. Thus, five centromeric satellite repeats exhibit distribution bias within the two subgenomes in FO accession. Scale bars: 1 μm.


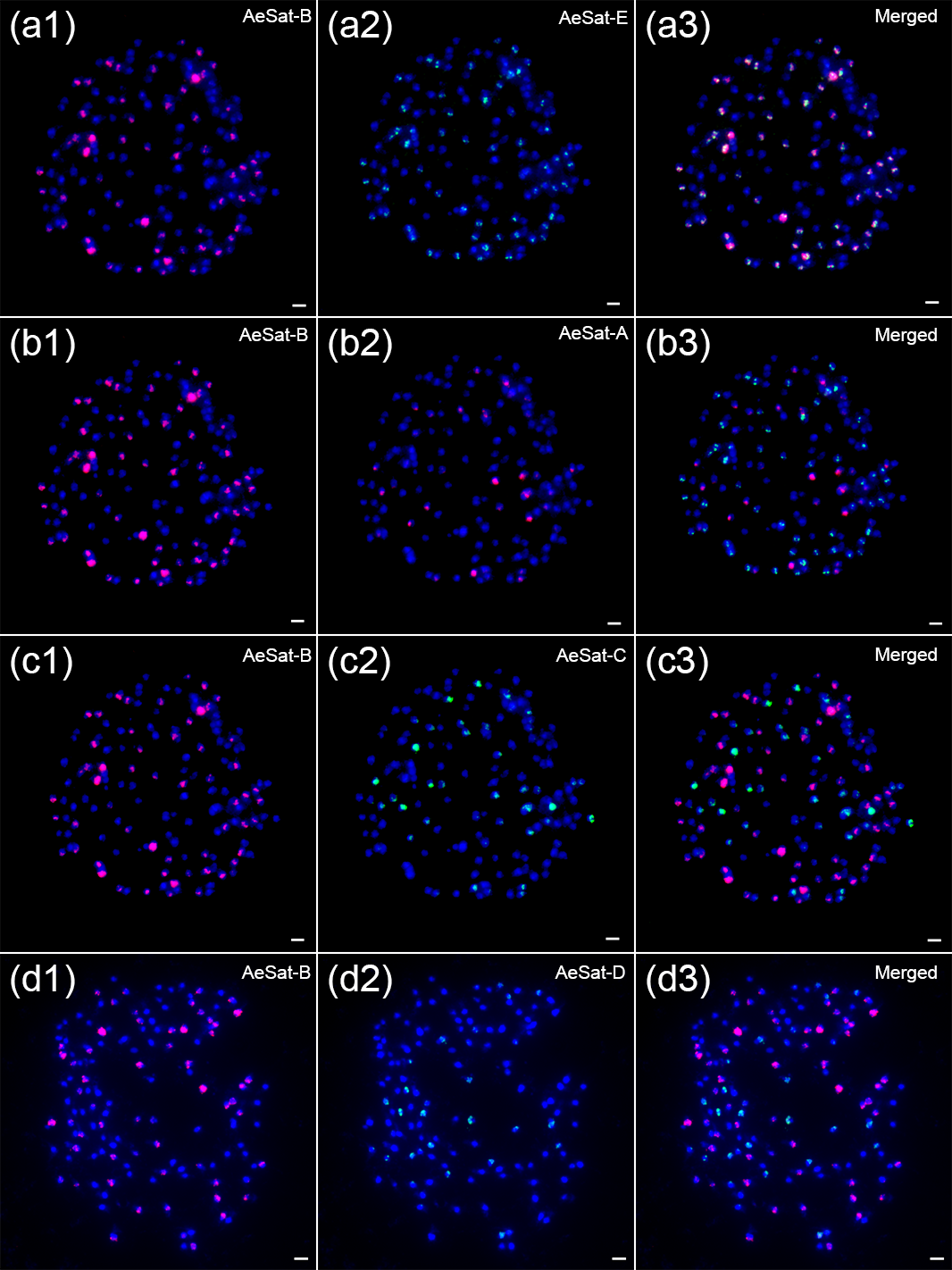


Figure S3. FISH localization of five centromere satellite repeats on mitotic metaphase chromosomes of T3 accession. (a) Dual-color FISH of AeSat-B (red) and AeSat-E (green), showing colocalization of these two satellite repeats on the chromosomes of T3; (b) Dual-color FISH of AeSat-B (red) and AeSat-A (green), showing non-colocalization of these two satellite repeats on the chromosomes of T3; (c) Dual-color FISH of AeSat-B (red) and AeSat-C (green), showing non-colocalization of these two satellite repeats except on one pair of chromosomes of T3; (d) Dual-color FISH of AeSat-B (red) and AeSat-D (green), showing colocalization of these two satellite repeats on a few chromosomes of T3. Thus, five centromeric satellite repeats exhibit distribution bias within the two subgenomes in T3 accession. Scale bars: 1 μm.


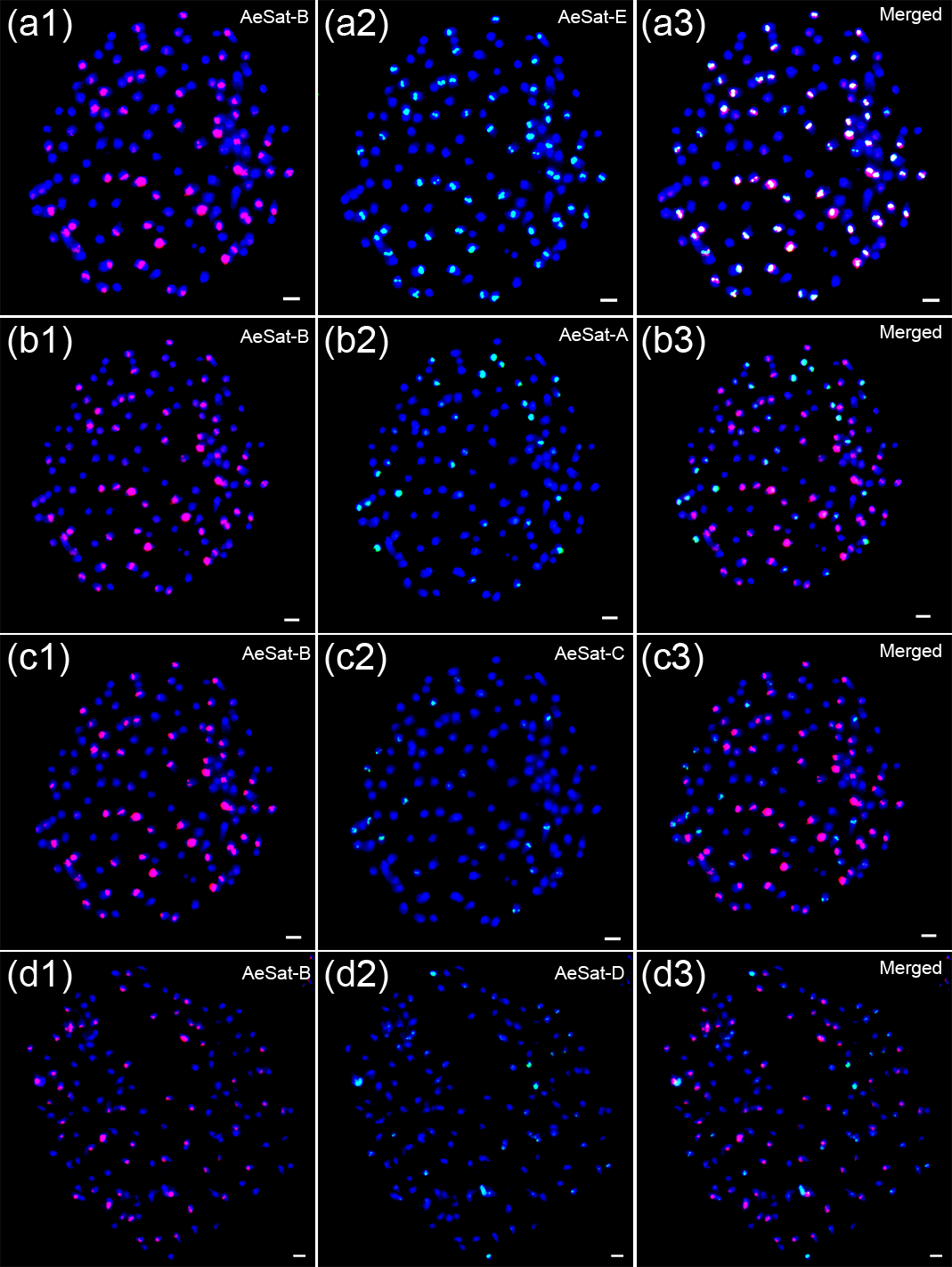


Figure S4. FISH localization of five centromere satellite repeats on mitotic metaphase chromosomes of ROJ accession. (a) Dual-color FISH of AeSat-B (red) and AeSat-E (green), showing colocalization of these two satellite repeats on the chromosomes of ROJ; (b) Dual-color FISH of AeSat-B (red) and AeSat-A (green), showing non-colocalization of these two satellite repeats on the chromosomes of ROJ; (c) Dual-color FISH of AeSat-B (red) and AeSat-C (green), showing non-colocalization of these two satellite repeats except on one pair of chromosomes of ROJ; (d) Dual-color FISH of AeSat-B (red) and AeSat-D (green), showing colocalization of these two satellite repeats on a few chromosomes of ROJ. Thus, five centromeric satellite repeats exhibit distribution bias within the two subgenomes in ROJ accession. Scale bars: 1 μm.


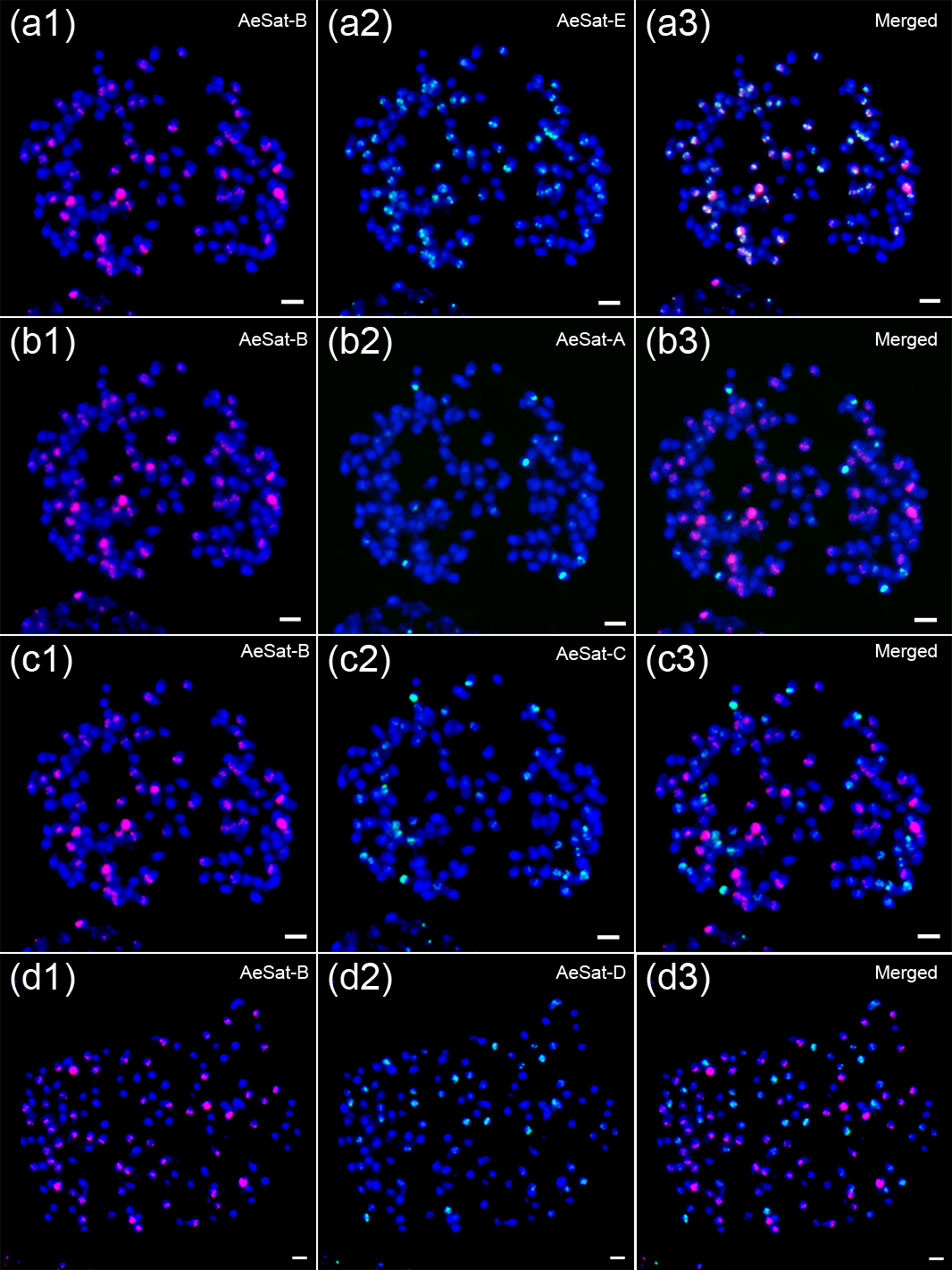


Figure S5. FISH localization of five centromere satellite repeats on mitotic metaphase chromosomes of QR accession. (a) Dual-color FISH of AeSat-B (red) and AeSat-E (green), showing colocalization of these two satellite repeats on the chromosomes of QR; (b) Dual-color FISH of AeSat-B (red) and AeSat-A (green), showing non-colocalization of these two satellite repeats on the chromosomes of QR; (c) Dual-color FISH of AeSat-B (red) and AeSat-C (green), showing non-colocalization of these two satellite repeats except on one pair of chromosomes of QR; (d) Dual-color FISH of AeSat-B (red) and AeSat-D (green), showing colocalization of these two satellite repeats on a few chromosomes of QR. Thus, five centromeric satellite repeats exhibit distribution bias within the two subgenomes in QR accession. Scale bars: 1 μm.


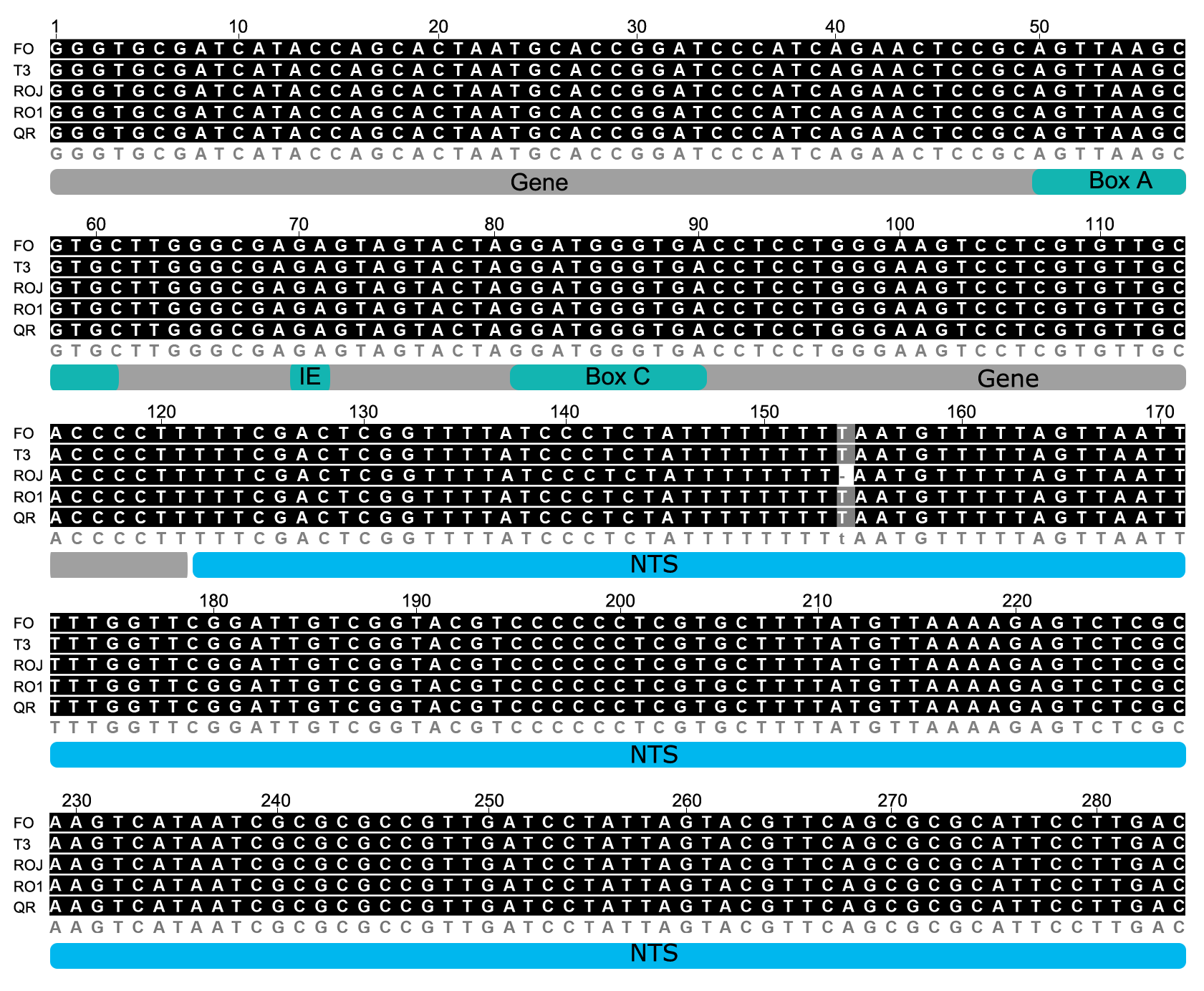


Figure S6. Multiple sequence alignment of 5S rDNA and NTS sequence from five okra accessions. Regions of the 5S rDNA and NTS sequence are shaded in the grey and blue box, respectively. The A-, IE- and C-Boxes of pol III promoters are marked in green.


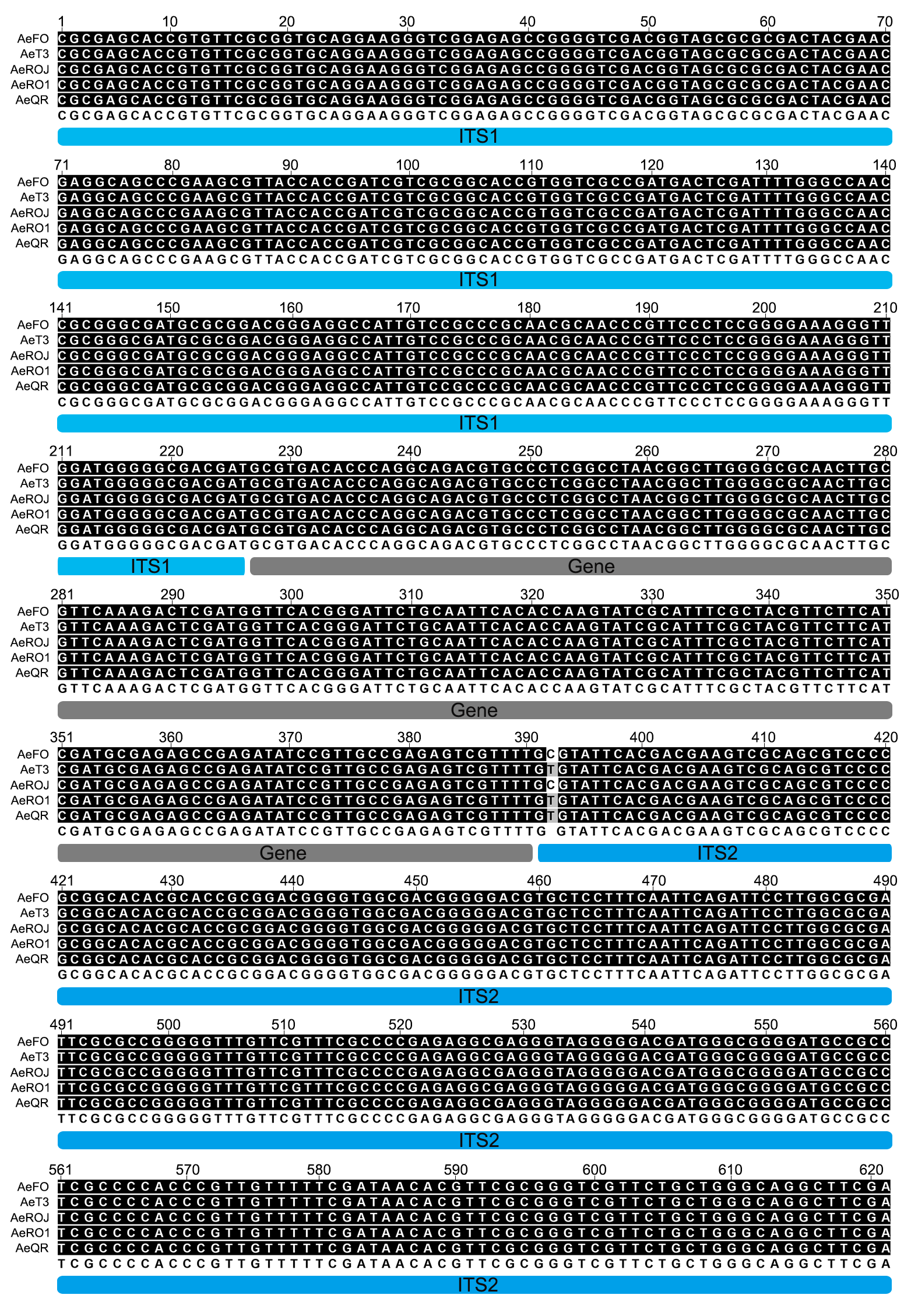


Figure S7. Multiple sequence alignment of ITS1+5.8S+ITS2 sequence from five okra accessions. Regions of the 5.8S rDNA as well as ITS1 and ITS2 sequence are shaded in the grey and blue box, respectively.


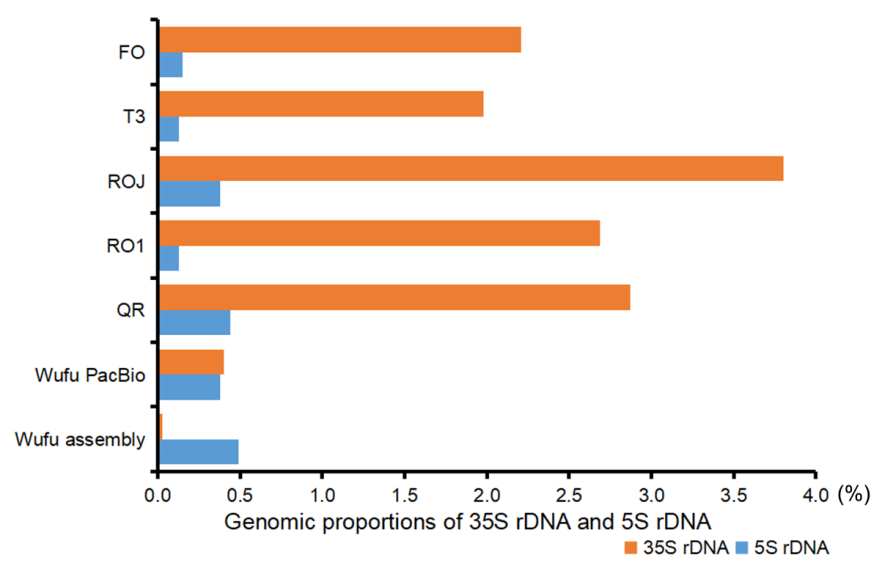


Figure S8. The genomic proportions of 35S rDNA and 5S rDNA in NGS, TGS and genome assembly.


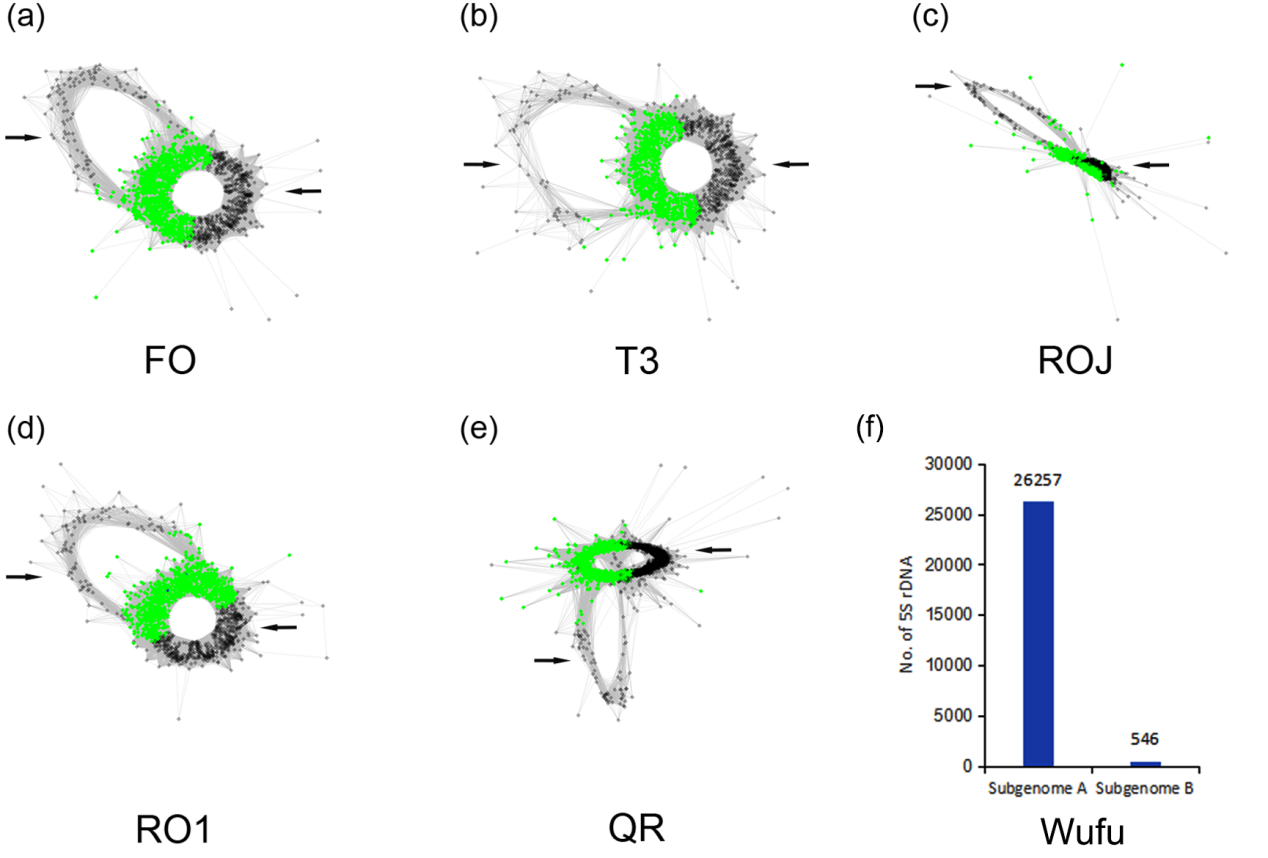


Figure S9. The 5S rDNA sequence reads organized in graph structures from the RepeatExplorer2 graphical output. (a-e) Single reads are represented by vertices (nodes) and their sequence overlaps by edges. The 5S rDNA coding sequences and intergenic spacers are highlighted in green and grey vertices, respectively. Two loop structures were interconnected by a junction region (annotated as the coding sequence of 5S rDNA) in five okra accessions. Arrows indicate two intergenic spacers of 5S rDNA. (f) The copy number of 5S rDNA in both subgenomes of the okra variety ‘Wufu’ genome assembly.


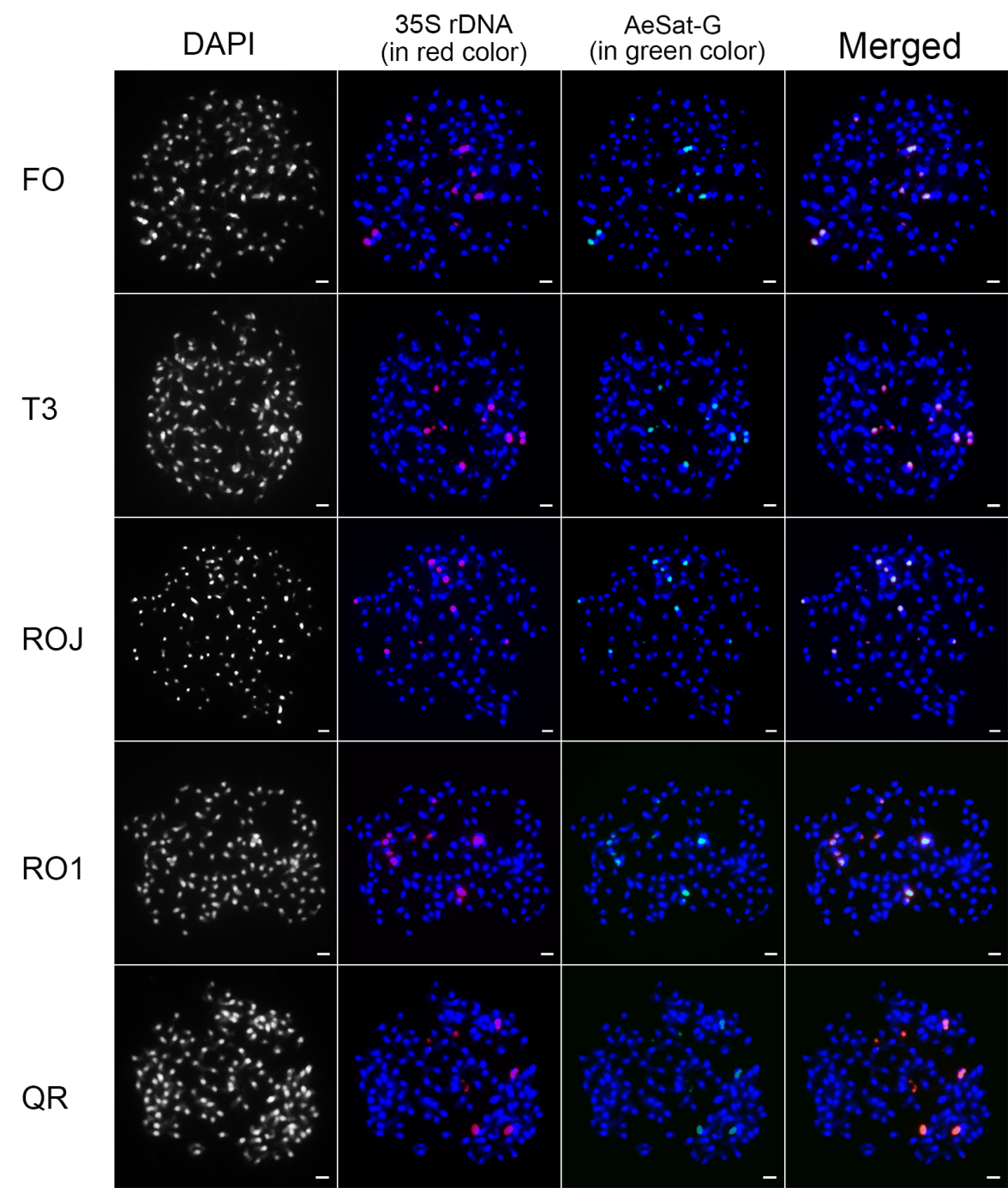


Figure S10. FISH mapping of 35S rDNA and AeSat-G on the metaphase spreads of the five okra accessions. DAPI-stained mitotic chromosomes are shown in blue and grey. The signals of 35S rDNA and AeSat-G are shown in red and green, respectively. (a1-a4) FISH mapping of 35S rDNA and AeSat-G in FO. (b1-b4) FISH mapping of 35S rDNA and AeSat-G in T3. (c1-c4) FISH mapping of 35S rDNA and AeSat-G in ROJ. (d1-d4) FISH mapping of 35S rDNA and AeSat-G in RO1. (e1-e4) FISH mapping of 35S rDNA and AeSat-G in QR. AeSat-G exhibited a propensity for localizing in regions with relatively low DAPI staining, and it co-localized with the 35S rDNA at the same chromosomal location. Scale bars: 1 μm.


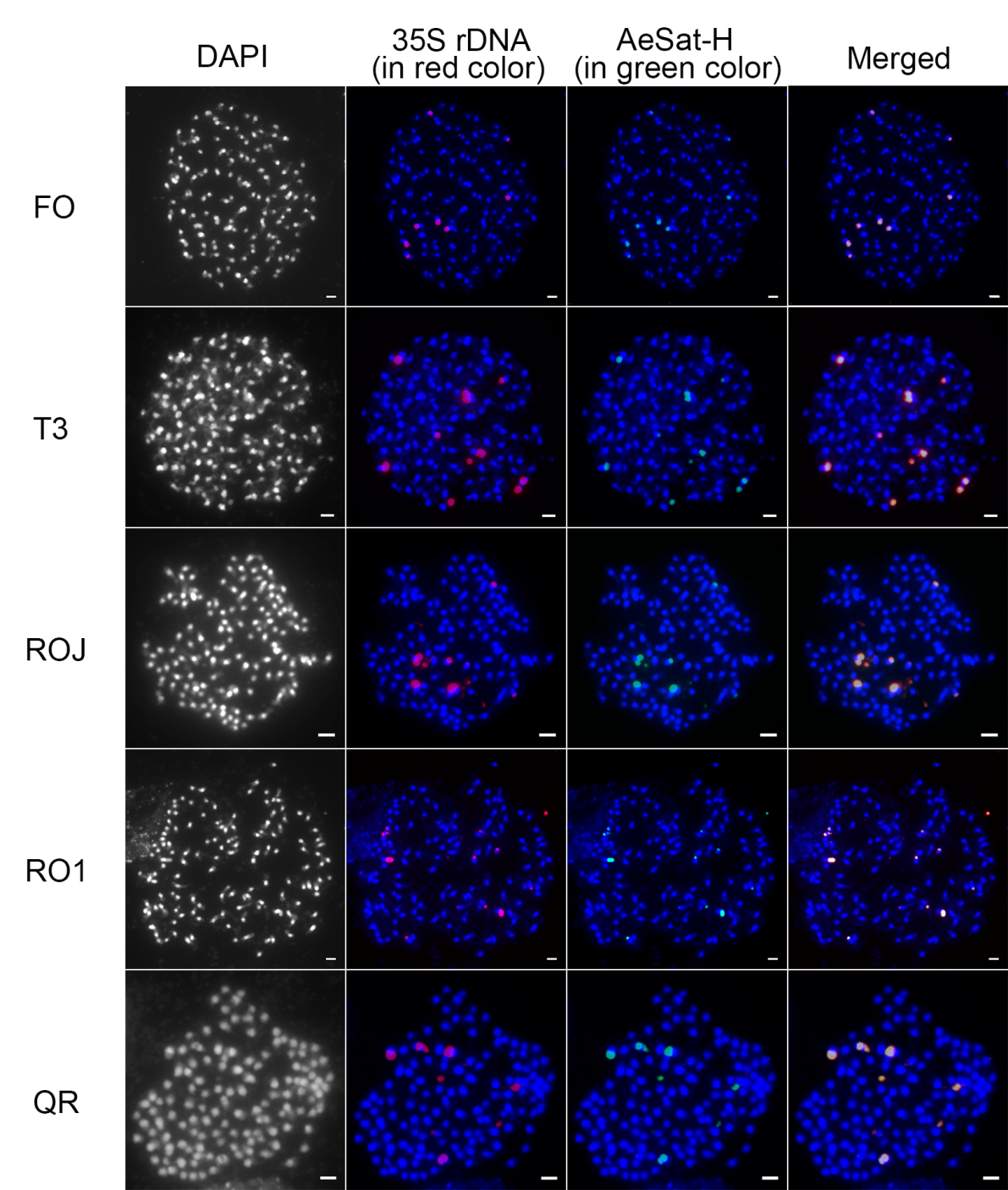


Figure S11. FISH mapping of 35S rDNA and AeSat-H on the metaphase spreads of the five okra accessions. DAPI-stained mitotic chromosomes are shown in blue and grey. The signals of 35S rDNA and AeSat-H are shown in red and green, respectively. (a1-a4) FISH mapping of 35S rDNA and AeSat-H in FO. (b1-b4) FISH mapping of 35S rDNA and AeSat-H in T3. (c1-c4) FISH mapping of 35S rDNA and AeSat-H in ROJ. (d1-d4) FISH mapping of 35S rDNA and AeSat-H in RO1. (e1-e4) FISH mapping of 35S rDNA and AeSat-H in QR. AeSat-H exhibited a propensity for localizing in regions with relatively low DAPI staining, and it co-localized with the 35S rDNA at the same chromosomal location. Scale bars: 1 μm.
